# Man and the Last Great Wilderness: Human Impact on the Deep Sea

**DOI:** 10.1371/journal.pone.0022588

**Published:** 2011-08-01

**Authors:** Eva Ramirez-Llodra, Paul A. Tyler, Maria C. Baker, Odd Aksel Bergstad, Malcolm R. Clark, Elva Escobar, Lisa A. Levin, Lenaick Menot, Ashley A. Rowden, Craig R. Smith, Cindy L. Van Dover

**Affiliations:** 1 Institut de Ciències del Mar, Consejo Superior de Investigaciones Científicas, Barcelona, Spain; 2 School of Ocean and Earth Science, University of Southampton, National Oceanography Centre Southampton, Southampton, United Kingdom; 3 Institute of Marine Research, Flødevigen, Norway; 4 National Institute of Water and Atmospheric Research, Wellington, New Zealand; 5 Universidad Nacional Autónoma de México, Instituto de Ciencias del Mar y Limnología, México, D.F., Mexico; 6 Integrative Oceanography Division, Scripps Institution of Oceanography, La Jolla, California, United States of America; 7 Ifremer, Brest, DEEP/LEP, Plouzane, France; 8 Department of Oceanography, University of Hawaii, Honolulu, Hawaii, United States of America; 9 Division of Marine Science and Conservation, Nicholas School of the Environment, Duke University, Beaufort, North Carolina, United States of America; California Academy of Sciences, United States of America

## Abstract

The deep sea, the largest ecosystem on Earth and one of the least studied, harbours high biodiversity and provides a wealth of resources. Although humans have used the oceans for millennia, technological developments now allow exploitation of fisheries resources, hydrocarbons and minerals below 2000 m depth. The remoteness of the deep seafloor has promoted the disposal of residues and litter. Ocean acidification and climate change now bring a new dimension of global effects. Thus the challenges facing the deep sea are large and accelerating, providing a new imperative for the science community, industry and national and international organizations to work together to develop successful exploitation management and conservation of the deep-sea ecosystem. This paper provides scientific expert judgement and a semi-quantitative analysis of past, present and future impacts of human-related activities on global deep-sea habitats within three categories: disposal, exploitation and climate change. The analysis is the result of a Census of Marine Life – SYNDEEP workshop (September 2008). A detailed review of known impacts and their effects is provided. The analysis shows how, in recent decades, the most significant anthropogenic activities that affect the deep sea have evolved from mainly disposal (past) to exploitation (present). We predict that from now and into the future, increases in atmospheric CO_2_ and facets and consequences of climate change will have the most impact on deep-sea habitats and their fauna. Synergies between different anthropogenic pressures and associated effects are discussed, indicating that most synergies are related to increased atmospheric CO_2_ and climate change effects. We identify deep-sea ecosystems we believe are at higher risk from human impacts in the near future: benthic communities on sedimentary upper slopes, cold-water corals, canyon benthic communities and seamount pelagic and benthic communities. We finalise this review with a short discussion on protection and management methods.

## Introduction

### From exploration to exploitation

Deep-sea exploration began a little over 150 years ago, initially promoted by the 19^th^ century debates on whether life occurred at depths below 300 m [1].The deep sea is considered to start at about 200 m depth, at the shelf break, where a clear change of fauna from shallow to deep water is observed [2]. The waters deeper than 200 m form the largest environment on Earth with a volume of 1368×10^6^ km^3^ covering an area of 360 million km^2^, equivalent to about 50% of the surface of the Earth, and have an average depth of 3800 m, with a maximum depth of 10,924 m in the Mariana Trench. Although the first record of a deep-sea species, the ophiuroid *Gorgonocephalus caputmedusae* (Linnaeus, 1758) (as *Astrophyton linckii*, Müller & Troschel, 1842), was provided by Sir John Ross in 1818 while sounding at 1600 m in the Northwest Passage [3], robust evidence of deep-sea fauna accumulated only from 1850. Life was found at bathyal depths in Norwegian fjords by Michael and Georg Ossian Sars and subsequently in abyssal waters (from 3000 to 6000 m) by Charles Wyville-Thomson during the cruises of HMS *Lightning* and HMS *Porcupine*. The celebrated worldwide cruise of HMS *Challenger* (1872–1876) found animals on all abyssal plains that were sampled. This expedition opened a period of national deep-sea exploration that culminated in the *Galathea* expedition of 1950–1952, which showed that animals live at all depths, including the deepest parts of the ocean. At the end of this period of pioneering exploration, our understanding of the deep ocean was one of low biodiversity, no primary production, no seasonality and a uniformly cold, food-poor, dark, tranquil and invariant environment. It was with this scientific framework that the United Nations Convention on the Law of the Sea (UNCLOS) was written and signed in 1972, and the deep-sea floor of the high-seas was deemed exploitable for biological resources and sea-floor minerals.

However, this view changed substantially in the following decades. In the late 1960s and 1970s, increasingly sophisticated sampling methodologies with the ability to collect quantitative samples of macrofauna demonstrated that the deep sea was much more biologically diverse than originally thought. In 1967, Hessler and Sanders [4] documented remarkable levels of species diversity in the deep sea, with up to 365 species in a single macrofaunal sample. Subsequently, in 1992, Grassle and Maciolek [5] estimated that the entire deep sea might contain up to 10 million species of small invertebrates, mostly polychaetes, peracarid crustaceans and molluscs (twice as many as the estimated 5 million species in rain forests [6]). This estimate generated considerable debate concerning the order of magnitude of species diversity [7], [8], but the general concept that the deep sea was a highly species-rich environment was now supported by intensive sampling efforts and rigorous statistical analyses [9]. Although deep-sea species have not proven to be eurytopic (i.e. able to adapt to a wide range of environmental conditions), they may show no more stenotopy (i.e. ability to adapt only to a narrow range of environmental conditions) than is found in shallow water [10]. Thus, regional diversity could be lower than originally anticipated and the most recent estimates of total deep-sea diversity of macrofauna are considerably less than 10 million species [11]. However, further detailed sampling and analyses are necessary to describe regional diversity patterns accurately.

The late 1970s and 1980s gave rise to many exciting discoveries in the deep sea, including hydrothermal vents [12], cold seeps [13], [14], chemosynthetic ecosystems created on whale falls [15], benthic storms [16] and seasonality [17]–[19]. With the greater use of remote techniques such as multibeam swath bathymetry and seafloor imagery, habitat heterogeneity in parts of the deep sea was shown to be high. This heterogeneity, taken together with the vast areas of the deep sea has reinforced the concept of high biodiversity [20]–[23].

By the end of the 20^th^ Century, the deep sea was recognised as the largest environment on Earth containing numerous sub-habitats, with unique abiotic and biological characteristics and supporting a particularly high biodiversity [24]. However, the deep sea has remained rather remote from public consciousness and the first exploitations and anthropogenic activities did not have any major social impact. The deep sea was (and still is) perceived as a service provider at two levels: (1) it served as a convenient site for disposal of waste, especially where land options were not politically and “ethically” attractive and (2) it was seen as a source of potential mineral and biological wealth over which there was no national jurisdiction. In the last decades, decreases in the amount of land-based and coastal resources combined with rapid technological development has driven increased interest in the exploration and exploitation of deep-sea goods and services, to advance at a faster pace than the acquisition of scientific knowledge of the ecosystems [25]–[27]. Evidence of this is found, for example, in the boom and bust cycle of many deep-sea fisheries in the 1970s–1980s [e.g. 28], [29], the disposal of sewage waste in deep water in the 1980s [30] and the dumping of chemical wastes and munitions [25]. Furthermore, human activities on land have promulgated a third and perhaps more dangerous level of impact: increasing atmospheric CO_2_ emissions that have resulted in climate change [31] – including the warming of the ocean, stratification and the generation and expansion of hypoxia – and ocean acidification [32]. A study by Halpern et al. [33] indicates that no area in the ocean is completely unaffected by anthropogenic impact and that most areas (41%) are affected by multiple drivers. Their model shows that coastal ecosystems receive the greatest cumulative impact, while polar regions and deep waters seem to be the least impacted [33]. Previous studies have reviewed different aspects of anthropogenic impact in the deep sea [25], [29], [34], [35], but to date little information is available on the direct and long-term effects of human activities in bathyal and abyssal ecosystems. The deep-water ecosystem is poorly understood in comparison with shallow-water and land areas, making environmental management in deep waters difficult. Deep-water ecosystem-based management and governance urgently need extensive new data and sound interpretation of available data at the regional and global scale as well as studies directly assessing impact on the faunal communities [27].

In this paper, we assess past, present and future impacts of human-related activities on deep-sea habitats and their communities, from disposal, through exploitation to climate change (including ocean acidification) using a semi-quantitative analytical approach. Studies on effects of anthropogenic impact on deep-sea habitats are still limited and often conducted at local or, at most, regional scales. We acknowledge this lack of global data and identify gaps that need urgent attention if we are to understand the resilience of deep-sea communities to anthropogenic stressors.

## Materials and Methods

### Semi-quantitative assessment of anthropogenic impacts in the deep sea

The traditional approach for quantitatively determining anthropogenic impacts in the marine environment is to conduct surveys before and after the impact takes place. This has proved difficult in the deep sea, as impact has often taken place before any baseline survey and the limited evidence to date suggests that the nature and extent of impacts can be variable [25], [29]. As a result, we have relied on the authors' collective and extensive experience of the deep-sea ecosystem together with the published literature, to provide a semi-quantitative scale of anthropogenic impact assessment. During the course of a Census of Marine Life SYNDEEP workshop (Sept. 2008), a group of 23 deep-sea researchers (see legend in Table S1) developed a scoring system to grade the effect of 28 major anthropogenic impacts grouped in 3 main categories (Table 1) on 12 deep-sea habitats (see description of habitats below). A first draft table of impact level was created, with the estimated impact level scored from 0 to 5 based on the discussions held during the workshop, for past, present and future impacts. These discussions reflect knowledge of the current literature and the experience and judgment of the researchers involved in this study. After the workshop, the draft table was circulated amongst all researchers for any further input and to achieve a final check and final consensus on the scores. Where insufficient information for an impact or an ecosystem led to uncertainty of impact level, a question mark (?) was used in the score. When there was no available evidence of an impact and the impact was unlikely, not applicable (NA) was used. We recognize that this scoring system is subjective, but in the absence of global quantitative data, it gives some indication of future impacts on the ecosystem services provided by the deep sea. Thus, allowing the economic and societal effects of anthropogenic impact to be considered.

**Table 1:** Main anthropogenic impacts considered in the semi-quantitative analysis (see Tables S1, S2 and S3) grouped under three main categories.

DISPOSAL	EXPLOITATION	OCEAN ACIDIFICATION & CLIMATE CHANGE
Clinker	Trawling	Ocean acidification
Sewage	Long-lining	Warming temperature
Dredge spoil	Ghost fishing	Hypoxia
Pharmaceuticals	Mining	Nutrient loading
Low-level radioactive waste	Oil and gas	Stratification
Radionucleids	Underwater cables	Deep circulation shutdown
Chemical contamination CFCs	Pipelines	Regional circulation change
Chemical contamination PAHs	Science	
Large structures (wrecks)	Acoustics	
Munitions		
CO_2_		

CFCs, Chlorofluorocarbons; PAHs, Polycyclic Aromatic Hydrocarbons.

We have conducted this analysis by dividing the deep sea according to the type of habitat. The main divisions may include several distinct sub-habitats and their characteristic (or at least best known) faunal components:

- Mid-ocean ridges, characterised by benthic sessile fauna and localised demersal and pelagic communities.
- Sedimentary slope (excluding other specific communities found on slopes such as cold-water corals, seeps, oxygen minimum zones), characterised by demersal fauna as well as epifaunal and infaunal benthos
- Canyons, with a high degree of habitat heterogeneity and diverse fauna varying with substratum: sessile benthos and demersal fauna characterize hard bottoms while mobile epifauna, infauna and demersal fauna abound in association with soft sediments.
- Seamounts, characterized by sessile benthos and abundant localised pelagic communities.
- Cold-water coral habitats, including the frame building corals and associated species.
- Active hydrothermal vents, characterised by benthic fauna with a high degree of endemicity.
- Cold seeps, characterised by benthic fauna with a relatively high degree of endemicity
- Oxygen minimum zones abutting margins, characterized by specialized benthic fauna.
- Abyssal plains, characterised by mobile epifauna and infauna.
- Manganese-nodule provinces, specific habitat on abyssal plains, characterised by sessile and mobile epifauna and infauna.
- Trenches, characterised by demersal megafauna and infauna.
- Bathypelagic water column, characterised by mid-water species.

For many of these environments there is little information about human impacts and stresses, so we have placed potential human impacts on the communities into the framework of local physico-chemical conditions. For example, hydrothermal vents inject as much trace metal volume into the deep ocean as the rivers of the world inject into coastal waters [36]. Thus, human impact on trace metal chemistry at vents through the disposal of metal ballast weights from submersibles is likely to be small, while it would be high in regions where no metals are present naturally.

#### Scaling

Anthropogenic impacts are seen, intuitively, as detrimental to deep-sea organisms at various scales. A specific impact will affect different habitats in different ways, depending on the abiotic characteristics of the habitat (geology, topography, biogeochemistry, currents) and biological variables such as community composition, existence of rare/endemic species, life history (lifespan, age at first maturity, gametogenesis, fecundity, larval type) of the species and their trophic relationships. An important factor affecting our capacity to score impact in the deep sea is our limited knowledge on biodiversity and ecosystem function for some habitats. All these variables (abiotic, biological and knowledge) were discussed and taken into account for each habitat in relation to each impact. The levels of impact were classified as follows:

- 5: major anthropogenic impact including death of all life at the point of impact. Likely to have subsequent regional effects.
- 4: major anthropogenic impact with very few species surviving with some or no regional effects.
- 3: moderate impact causing possible reduction in biodiversity and potential reduction in biomass and productivity on a local basis.
- 2: minor impact on fauna or habitat, partially cosmetic but not easily rectified.
- 1: minor impact on fauna or habitat, mainly cosmetic and relatively easily rectified.
- 0: no discernable impact or reduction/increase in biodiversity.
- N/A: impact not applicable to the ecosystem in question.
- ?: no evidence and unknown effect of impact.

Tables S1, S2 and S3 include the scaling for each individual impact in each habitat, the total and mean impact for each of the main categories (disposal, exploitation and climate change) in each habitat and a grand total and grand mean that include all impacts in each habitat for past, present and future respectively. The mean impacts for each category (disposal, exploitation and ocean acidification/climate change) were calculated as the total impact for the category considered divided by the number of individual impacts within that category. The grand total and grand mean impacts have been coded with bold and italics to highlight the ecosystems at higher risk (Tables S1, S2 and S3). These tables are intended to be modified as we understand more precisely anthropogenic impact on the deep sea.

Impact scores in Tables S1, S2 and S3 are given for impacts in isolation. We then progressed to consider the combined or simultaneous effect of interactions amongst different impacts. For this, an interaction matrix was created, where 1 designates the presence of an interaction between two impacts and 0 designates the absence of such interaction. A figure was created to illustrate the major synergies amongst the different anthropogenic impacts considered.

## Results and Discussion

### Disposal of litter and waste

The deep seafloor is, for most people, out of sight and therefore, often, out of mind. This has encouraged, for centuries, the dumping of waste of all sorts into deep waters, with (largely) unknown and un-studied effects on the habitats and their fauna. Although dumping waste and litter into the sea is now legally banned, the problem persists because of the historical accumulation of marine litter in all the world's oceans.

#### Over the side of ships – marine litter

**Figure 1:**
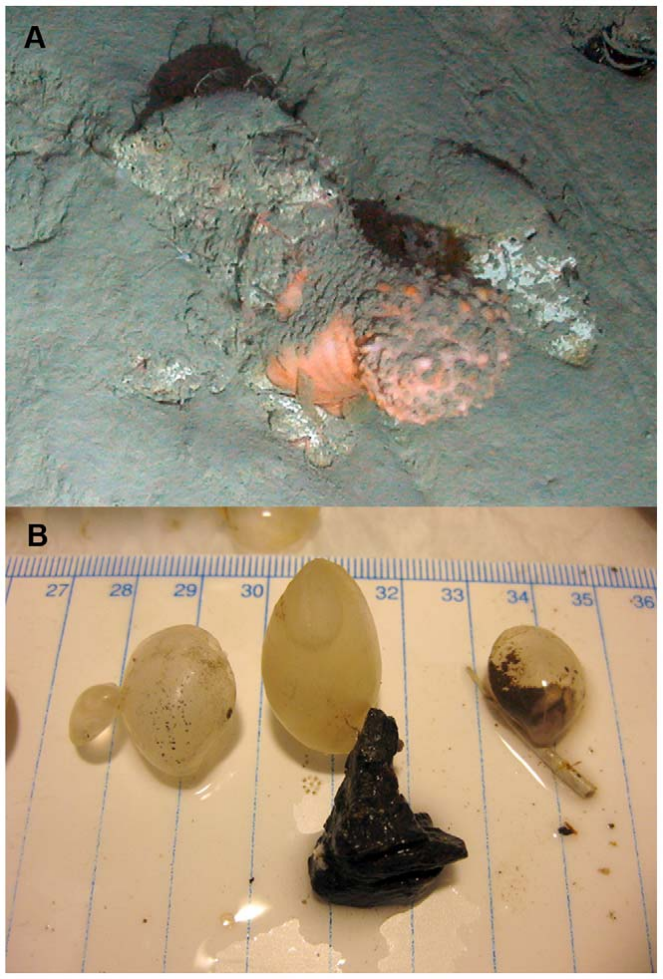
Deep-water fauna attached to clinker. A, the anemone *Phelliactis robusta*, from 2311 m in the Eastern Whittard Canyon, SW Ireland, taken during cruise JC10, Dive 65, of the HERMES project (Photo courtesy of P. Tyler, Uni. Southampton, and D. Masson, NOCS/NERC); B, the brachiopod *Gryphus vitreus* attached to clinker and to a scaphopod shell (Photo courtesy of Ariadna Mechò, ICM-CSIC).

The United Nations Environment Programme (UNEP) defines marine litter as “any persistent, manufactured or processed solid material discarded, disposed of or abandoned in the marine and coastal environment”. The first intended (as opposed to accidental) disposal of waste in the deep sea predates scientific interest in this environment. The age of sail gave way to the age of steam at the end of the 18^th^ century and, for the next 150 years, one of the main waste products of steam power was a hard residue of burnt coal called clinker. This material was usually dumped over the ship's side. In a survey on the nodule-free abyssal plain in the northeastern Atlantic, Kidd and Huggett [37] showed that clinker formed more than 50% of the hard substratum (the other being glacial drop stones) and that this clinker formed a suitable attachment point for the anemone *Phelliactis robusta* [38] (Figure 1A), although it appears toxic to other deep-sea species. On the northwestern Mediterranean margin, clinker can provide common substratum to the brachiopod *Gryphus vitreus* (Figure 1B), but otherwise this substratum is not colonized by sessile metazoan species. In the past, clinker has been disposed of on abyssal plains, sedimentary slopes and in some canyons (Table S1). Major occurrences of clinker may be found off large ports where steamships cleaned their boilers (Tyler pers. obs.). Clinker is no longer dumped into the ocean because steam power is no longer used over the deep ocean and modern regulations would prevent its disposal. Thus, the impacts of clinker in providing hard substratum are stable or declining with sediment accumulation.

**Figure 2:**
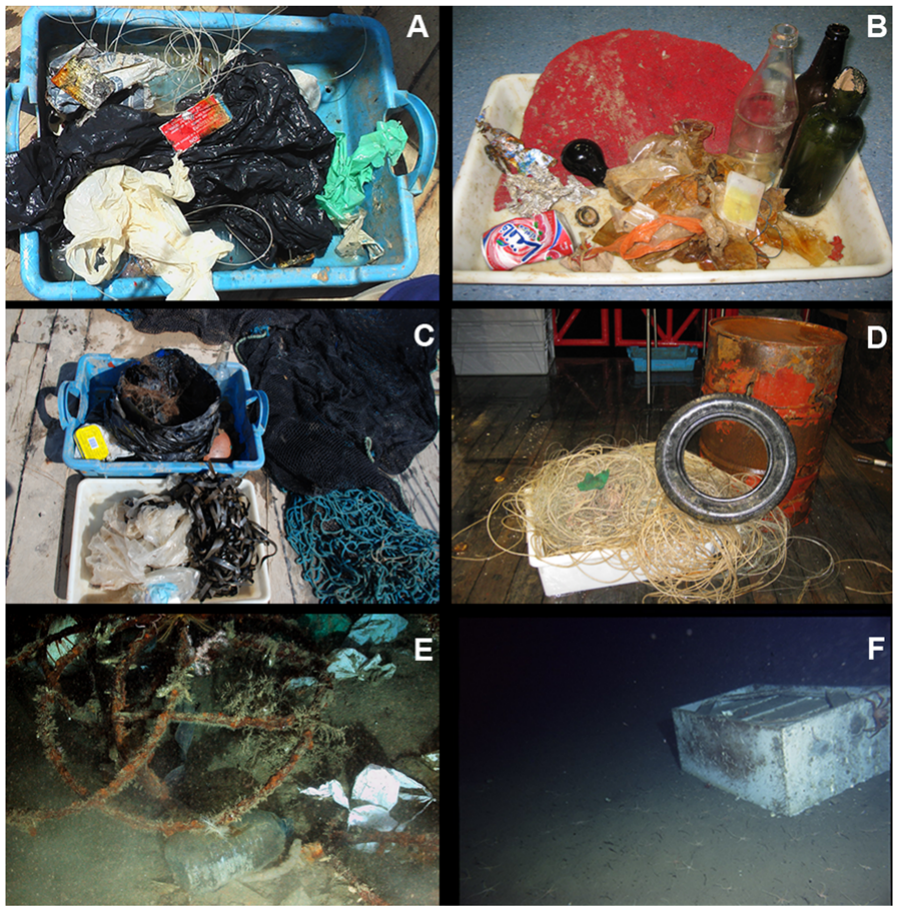
Litter observed and collected from bathyal and abyssal depths. A–C, litter collected from the Western Mediterranean at 1200 m (A), 2000 m (B) and 3000 m (C) (Photos courtesy of E. Ramirez-Llodra, ICM-CSIC); D, oil drum, tyre and longline collected from the Central Mediterranean at 1200 m depth (Photos courtesy of E. Ramirez-Llodra, ICM-CSIC); E, litter observed with the ROV Isis in the Lisbon canyon (Photo courtesy of P. Tyler, Uni. Southampton/NOCS); F, litter observed with a submersible on the southern California margin at 1240 m depth (Photo courtesy of C. Smith, Uni. Hawaii).

**Figure 3:**
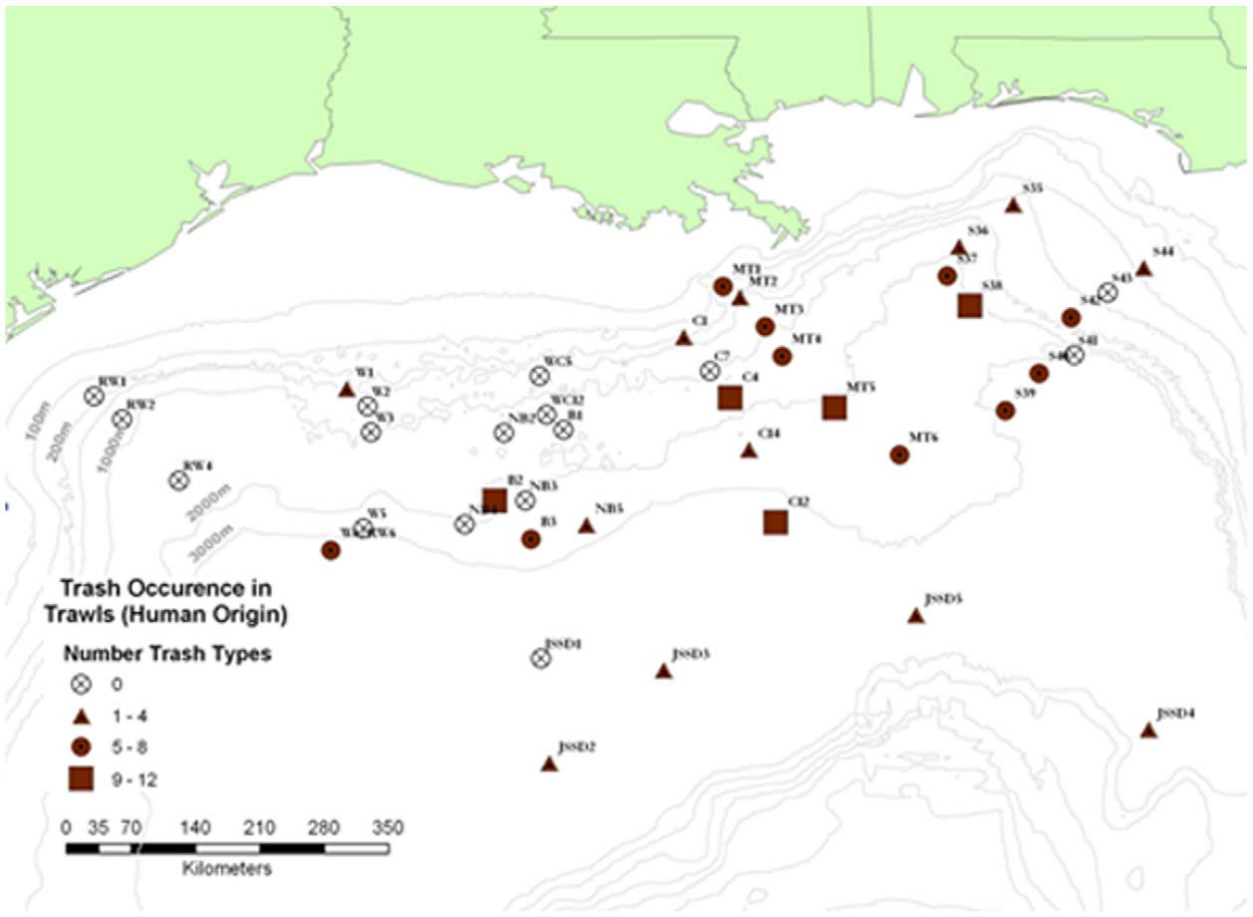
Litter occurrence at bathyal and abyssal depths in the Gulf of Mexico. Image courtesy of Gilbert Rowe, MMS contract 30991, Figure 8.7.1 of the DGoMB report (from Rowe and Kennicutt, 2009).

The routine dumping of many types of waste from ships was legally banned from 1972 onward (London Convention, 1972). A new and stricter convention was negotiated in 1996, but did not enter into force until 2006 (http://www.imo.org). Before the ban, a large variety of litter was dumped from ships in transit, including from bulk carriers, tankers, fishing boats, ferries and yachts. The amount of litter dumped in the oceans from vessels each year is estimated to exceed 636,000 tonnes [39]. At present, litter continues to accumulate, through illegal disposal of litter from ships and lost or discarded fishing gear, as well being advected from the coast and river discharges [40]. Approximately 6.4 million tonnes per year of litter are dumped into the oceans [41], part of which sinks to bathyal and abyssal depths. Highly erosive deep-sea storms, which may affect 10% of the deep-sea floor, can transport laterally sediment loads along with benthic fauna [42]. It is reasonable to assume that these storms may also transport refuse to seafloor depressions, which can serve as debris traps. As sediments move down slope, they form debris flows and turbidity currents [43], which may work as an additional transport mechanism. Wood construction material and scraps of wood, bark, macrophytes and fruit that provide both habitat and nourishment to marine organisms [44]–[46] have been documented in deep-sea trawl samples periodically for at least three decades [47]–[49]. No definitive quantitative documentation, however, yet allows generalizations to be made about human generated refuse in deep-sea environments [50]. Litter is observed in almost all scientific seafloor surveys using video (e.g. remote operated vehicles, ROVs) and trawls. However, the amount of litter varies in different regions and no dedicated studies have been conducted to estimate the extent of litter accumulation in deep-sea habitats or to assess the effect of different litter types in the habitat and its effects on the fauna [51]. The most common litter types found on the deep–sea floor in the Mediterranean and northeastern Atlantic are soft plastic (e.g. bags), hard plastic (e.g. bottles, containers), glass and metal (e.g. tins, cans) (Figure 2A–C) [24], [52]–[55]. As part of a project investigating the biodiversity of bathyal and abyssal Mediterranean environments, 20 trawls were conducted using an otter trawl, covering a total area of 1 km^2^. Of these trawls, two collected an oil drum, and this is not uncommon (Ramirez-Llodra, pers. obs.) (Figure 2D). Furthermore, a study of the Blanes margin (northwestern Mediterranean) between 900 and 1500 m depth has shown that litter accumulates in the deepest areas sampled (Ramirez-Llodra, unpublished data). Conversely, careful examination of the Lisbon, Setúbal, Nazaré and Whittard canyon systems of the northeastern Atlantic by ROV showed only minor litter with the majority in the Lisbon Canyon off the Tagus mouth (Paul Tyler, pers. obs.) (Figure 2E). Observations from submersibles at depths of 1000–2000 m on the southern California margin reveal that litter, in the form of torpedo wire, plastic bags and miscellaneous items (shoes, furniture, naval debris, etc.) is the primary source of solid substrata at bathyal depths in this region (CR Smith, pers. obs. from about 50 submersible and ROV dives) (Figure 2F). A recent study by [56] reported the distribution, abundance and composition of litter at depth along the U.S. West Coast and found that plastics and metals were the most common types. Miyake et al [55] have used the JAMSTEC online deep-sea image database to conduct analysis of the occurrence and type of marine litter in the deep waters off Japan. The deepest litter to be observed was a waste can at 7216 m in the Ryukyu Trench. The canyons and abyssal plain off canyons often function as debris traps [53], an example being the Mississippi River Trough. A study of this area showed that trawls at 25 of the 34 sites (74%) contained human generated refuse dominated by plastics, aluminium cans, wood, and fishing gear [57] (Figure 3). Some continental margins, however, remain relatively uncontaminated, although even the most remote margins are not immune from impact. During 30 tows (roughly 18 km length) at 600 m depth along the Antarctic Peninsula with a 6.5 m otter trawl [58], litter collected consisted of two metal cans (CR Smith pers. obs.).

Since the mass production of plastics began about 60 years ago, the use of this long-lasting cheap material has increased and, in parallel, so has its waste. Plastics are found everywhere, from land to the oceans, from the coast to the deep sea [59]. Although some types of litter are recognizable, there is accumulating evidence that “mermaids' tears” (5 mm in diameter) and microplastics (microscopic sand grain-sized particles of eroded plastic) are becoming more common in the world oceans, including the deep sea [60], [61]. While the standing stock of mega- and macro-plastics in the oceans seems to be relatively stable, the size of plastic debris is decreasing and the amount of microplastics is increasing as a function of larger plastic breakdown and an increase in primary microplastics. In recent years, the use of biodegradable materials was proposed as a solution to the accumulation of plastics in the environment, but in some cases, the degradable material merely disintegrated into smaller pieces that are not degradable [58]. Little is known, however, of the true effect of these particles on the environment and the fauna [62]. Several studies have shown that effects such as ingestion by invertebrates could facilitate the transport of hydrophobic contaminants [63] and the release of potentially toxic bisphenol A and PS oligomers during plastic breakdown, which can disrupt hormonal functioning and reproductive systems in the fauna [64]. Studies in the deep sea are practically nonexistent and an urgent assessment of the impact of microplastics on deep-sea fauna is needed along with the development of methods to quantify and monitor their abundance and to identify potential sources and sinks of this debris.

Scientific research using moorings, submersibles and ROVs also contributes to deep-sea litter by the dropping of ballast weights (often solid plates or pellets of mild steel), although these contributions are small in relation to other sources of litter. On dives where there may be rock sampling, up to 65 kg of descent weight may be discarded at the seabed per dive (D. Turner pers. com.). For of submersibles, almost one tonne of weight is discarded per dive (Y. Hublot, pers. com.). At intensively studied sites, this can lead to a significant accumulation. In addition, instruments such as current-meter moorings, markers and other scientific tools are placed on the seabed and recovered after variable periods. When recovered, moorings leave their ballast behind, which can consist of steel (e.g. train wheels), lead or cement. This may be an issue in areas where there are recurrent investigations, such as the abyssal Gulf of Mexico, where mooring deployments for a single programme of physical oceanography baseline studies occur twice a year, leaving one tonne of iron per mooring and per year. Furthermore, a small proportion of these instruments are unrecoverable and lost at sea.

Most data available on marine litter are the by-product result of other projects targeting fauna [54], [65] and there are no standardized quantification methods. Impacts of litter on deep-sea habitats and fauna may include suffocation of animals from plastics, release of toxic chemicals, propagation of invasive species, physical damage to sessile fauna such as cold-water corals from discarded fishing gear, and ghost fishing from lost/discarded nets [59], [66], but these impacts are poorly quantified on a large geographical scale. The increasing evidence of continuous accumulation of litter has been recognised by the UNEP-Regional Seas initiative, which identified the need for further research on the impacts of marine litter in coastal areas [41] and with an increasing interest in deep-sea habitats (Gjerde, pers. com.). Current international multidisciplinary research programmes, such as the EU funded HERMIONE (Hotspot Ecosystem Research and Man's Impact on European Seas) project that investigates deep-sea ecosystem function and its contribution to production of goods and services, are incorporating studies of litter accumulation and impact. The Deep Gulf of Mexico Benthos Program recorded and classified refuse in the abyss [64] as well as chemical contamination in sediments [67] and fauna [68].

#### Sewage, dredge and mining waste

An example of significant but localized waste was the Deep Water dumpsite 106 at bathyal depths (about 2500 m) (sedimentary slopes) along the eastern seaboard of the United States. The site was used for the disposal of industrial and municipal wastes from 1972 [69] and continued to receive sewage until 1992. In 1981, Ohlhorst published a report describing the use of remote sensing to monitor ocean dumping at this site and showed plume dispersal with the widest cross-section of the plume measuring 2100 m [70]. By 1992, the dumpsite had received about 36 million tonnes of wet sewage sludge [25]. The sludge contained silver and persistent organic pollutants. Van Dover et al. [30] demonstrated incorporation of sewage-derived organic matter by benthic deposit feeders based on altered stable isotope ratios of megafauna, whilst Bothner et al. [71] showed that there were clear faunal changes at the seabed. It is highly likely that the bathypelagic ecosystem was affected in un-quantified ways as the dredge spoil was being dumped (Table S1). Following the cessation of dumping in 1992 there was an immediate improvement in local conditions, although indicators of pollution were still measurable at 75 km down-stream [25]. There is no known dumping of sewage or dredge spoil in the deep sea at present or planned for the future, although the option has been discussed for decades [72].

In addition, deep-sea disposal of terrestrial mine tailings is a future problem for island nations, such as Papua New Guinea, where access to deep water via pipelines is feasible. Deep disposal of toxic mining waste generated on land occurs from Lihir Gold on Lihir Island (New Ireland, Papua New Guinea), and is being considered by a copper mine in Papua New Guinea also (A. Harris, T. Nonggorr, pers. com.).

#### Fishing waste

Fishing vessels can produce waste products in addition to those considered above. Discharged processing waste from factory trawlers (e.g., fish heads, guts, frames), as well as whole fish that are lost or discarded at the surface, can affect other animals. In the early years of fishing for orange roughy off New Zealand, catches were often too large to be hauled back on board, and nets would burst spilling tens of tonnes of fish [e.g. 73], [74]. Seabird populations can benefit substantially from foraging on offal and discards [e.g. 75], [76] but the attraction of both seabirds and marine mammals (in particular seals) has become an important issue worldwide. Although they may benefit from increased food, they are also at risk from lines, hooks and wires, and can drown when trapped in trawls or taking bait or catch off longline hooks. This is an on-going problem for responsible fisheries, leading to devices such as bird-scarers, seal-exclusion grids, and regulations governing the discharge of offal. Such offal discharge can reach considerable depths. Offal from a New Zealand fishery for hoki (*Macruronus novaezelandiae*) was reported to reduce oxygen levels at 800 m depth [77] and possibly alter benthic community composition [78]. However, management of offal can minimise upper ocean impacts with careful disposal (e.g., only at night and away from sensitive habitat such as seamounts), using mincers to grind up the heads and bodies into small pieces, or rendering the waste into fishmeal.

#### Dead animals in the oceans

Livestock transported on ships may die while at sea. When this occurs, the dead animals might be dumped into the ocean, contributing occasional large pulses of organic material to the seafloor, similar to other natural large-organic falls (e.g. whale falls, kelp falls). Whale falls deliver large pulses of organic material (40-tonne whale carcass as the typical amount) [79] to the seafloor, providing significant inputs of organic matter to the normally food-limited deep sea and being most prevalent along migration corridors of the dominant large whale species [80]. For example, hundreds of grey whales sink to the seafloor annually within an area of 8×10^5^ km^2^ along the eastern Pacific [15]. This density was similar or probably larger in the 19^th^ century, as depicted in the whale charts published by M.F. Maury [81], and whaling is predicted to have restricted the distribution of whale-fall colonists [82], [83]. Most animals that die at sea while being shipped die of scabby-mouth [84] and salmonellosis [85], and ‘slaughter at sea’ sometimes occurs [85]. Some vessels that carry large numbers of livestock are equipped with a macerator to grind animals that die on route, and then channel the remains straight into the sea [86]. In addition, there is evidence of diseased animals being killed and their carcasses being burnt and sunk at sea. For example, 70,000 sheep were dumped from a ship in the Indian Ocean in 1996. Another 10,000 sheep were dumped from a ship en route between New Zealand and Saudi Arabia in 1990, and 40,000 off the coast of southern Australia [86], [87]. In July 2002, about 270,400 sheep died and where dumped at sea while en route to the Middle East [88]. The Keniry Report (2003) [85] has acknowledged that with about 300,000 sheep and 10,000 cattle being transported by sea at any time, the potential dumping of deceased animals could create a significant environmental perturbation. No evidence of disease transmission from dead livestock to deep-sea communities has been evaluated or recorded to date.

#### Pharmaceuticals

There has been some intentional disposal of pharmaceuticals in the deep sea. One of the main disposal sites was the Puerto Rico Trench. Prior to the 1980s, Puerto Rico gave tax advantages to pharmaceutical companies and their waste material was dumped in the trench at about 6000 m depth approximately 40 miles to the north of the island [89] (Table S1). Between 1973 and 1978, more than 387,000 tonnes of wastes were dumped in the trench (equivalent to 880 Boeing 747s) (http://deepseanews.com/2008/04/dumping-pharmaceutical-waste-in-the-deep-sea/). However, this dumping ceased in the early 1980s (Tables S2 and S3). Studies of the region used for waste disposal found demonstrable changes in the marine microbial community [90], [91]. Grimes et al. [92] found that *Pseudomonas* spp., reportedly common a decade earlier, were virtually absent from all samples taken from the dump site during a three year study, and an increase in *Staphylococcus* was evident. Nicol et al. [93] showed that pharmaceutical wastes disposed of in the Puerto Rico Trench were acutely toxic to many marine invertebrates. Laboratory studies demonstrated that tolerance between animals was variable, affecting survival rates, fecundity, adult size and normal growth. The amphipod *Amphithoe valida* suffered chronic toxicity in response to the dumped waste [94]. Antibiotics can have a negative impact on marine microorganisms, although the available evidence suggests that the impact for the slope and the pelagic fauna is low (Table S2).

At present there is no direct disposal of pharmaceutical products in the deep ocean. However, certain pharmaceuticals used by humans and livestock such as antibiotics, anti-depressants, birth control pills, cancer treatments and pain killers have been detected in various water sources and may pose a threat to the marine environment. Careless disposal of unused medicines can pass into waterways, as can human excreta containing incompletely metabolized medicines. Some of these drugs are non-biodegradable and are mutagenic, carcinogenic and teratogenic. Pharmaceutical wastes are an important issue for environmental management as they are so widely used, although their impact in the deep sea is uncertain. To date, there are limited studies of the impacts of pharmaceuticals on marine organisms and the few that exist have been conducted in shallow-water environments [e.g. 95 and references therein].

#### Low level radioactive waste and radionucleides

More controversial has been the disposal of radioactive waste in the deep sea. Anthropogenic radionuclides are often elevated in deep-sea sediment [96] and midwater organisms [97] but the discovery of radioactive elements in holothurians at 5000 m from weapons testing [98] was not readily explained until the understanding of vertical flux characteristics of surface-derived phytodetritus [99]. The disposal of radioactive waste has been much more difficult to monitor. Radioactive waste disposal has been concentrated on the slope and canyons of the northeastern Atlantic, and smaller disposals have occured in the northwestern Atlantic and the northeastern and northwestern Pacific [25] (Table S1). Most of the waste was stored in drums and tipped over the side of ships. Although there was an active programme in the 1980s to assess the feasibility and potential impacts of high-level radioactive waste disposal in the deep sea, political considerations stopped this programme and no intentional disposal of any radioactive waste occurs in the ocean today (Tables S1 and S2). Highly-focused sources of high level radioactive waste are associated with sunken nuclear submarines such as the U.S. submarines *Thresher* and *Scorpion* and the Russian submarine *Konsomalets*. Knowledge of the localized environmental impacts of these accidental sinkings is limited. Radiological monitoring of the U.S. submarines was undertaken in the years following their loss, but no significant environmental changes were observed [100], [101]. A study of the possible long-term release of radionuclides from the *Konosmalets* submarine indicated that the sunken submarine represented no significant threat to the environment [102]. Loss of nuclear submarines in deep water is a rare event although redundant nuclear submarines are stored in the shallow water of the Russian Arctic where their future impact is unknown.

#### Chemical contamination

Chemical contamination of deep-sea sediments and their effect on the fauna is still mostly unstudied. Although few studies are available, the recent increased sophistication of chemical analyses since the 2000s has shown that chemicals are accumulating in deep-sea sediments, benthos and midwater fauna (Table S2) [29], [103], [104]. The major contaminants of concern are persistent organic pollutants, toxic metals (e.g. Hg, Cd, Pb, Ni and isotopic tracers), radioelements, pesticides, herbicides and pharmaceuticals. Xenobiotics are chemicals found in an organism but are not normally produced or expected to be found in that organism. Some xenobiotics, such as synthetic organochlofoforides used in pesticides and plastics are resistant to degradation and deep-sea sediments have been suggested as the final accumulation site for these man-made pollutants [105]. Biochemical effects of xenobiotics (i.e., induction of cytochrome P450E that catalyzes transformation of foreign compounds) were first reported in rattail fish collected from depths greater than 1000 m [106], [107]. More recent work reinforces the view that organisms and sediments of the deep sea are global sinks for persistent semi-volatile contaminants [103], [104], with bioaccufmulation and enrichment in deep-sea organisms a consequence of consumption and recycling of pre-enriched organic matter as it sinks through the water column. Recent studies have provided evidence of low, but still toxic, levels of persistent organic pollutants such as polychlorinated biphenyls (PCBs) and dichlorodiphenyldichloroethylene (DDE) in sediments. Unger et al. [108] have shown elevated levels of persistent organic pollutants in nine species of cephalopod from mesopelagic and bathypelagic depths (1120 to 2980 m). Significant concentrations of persistent organic pollutants of industrial origin such as dioxins have been detected in the red shrimp *Aristeus antennatus* in the Western Mediterranean, where higher concentrations in the population from 2000 m than in that from 500 m depth [109]. Persistent organic pollutants have also been found in demersal fish between 900 and 1500 m depth on the Blanes margin, northwestern Mediterranean (S. Koenig, unpublished data). The bioaccumufolation of polycyclic aromatic hydrocarbons by amphipods in the deep Gulf of Mexico shows differences in the concentration of these compounds in the sediment and fauna, which suggest preferential uptake of certain compounds [68]. Hydrodynamics play a major role in the accumulation of chemical pollutants in deep-sea habitats. For example, climate-driven dense shelf water cascading events, such as the ones observed in the Gulf of Lions and other regions in the world, transport large amounts of sediment from the shelf and margin down to the lower slope and abyss [110], [111], where chemical contaminants can accumulate. Overall, the impact of persistent organic pollutants in the deep sea would appear to be low at present (Table S2), mainly because of dilution. It may be that only at the higher trophic levels are these contaminants concentrated enough to be toxic at present, but the accumulation of chemicals in deep waters and deep sediments, and the bioaccumulation in organisms might have a significant impact in the future.

#### Large structures

Ships have been lost since humans first took to the sea. In the Mediterranean, the most ancient wrecks are in shallow water and virtually nothing is known of any ships lost in deep water before the 20^th^ century. The sinking of ships contributed cannons and cannonballs as hard substratum and wood was integrated into the food webs. During the 20^th^ century, the loss of ships in the ocean was substantial, both in the number of vessels sunk and in their total tonnage. Between 1970 and 1990, the equivalent of 18 ships and 65,000 tons of shipping sank on the high seas (excluding coastal waters) per year [25]. Both world wars would have contributed considerably more than this in both merchant and military losses [35]. To illustrate the scale, in World War II, for example, during the battle of the Atlantic, more than 175 military ships were lost and more than 3500 merchant British ships (excluding other allies) were sunk, many of them in deep water [112] (http://en.wikipedia.org/wiki/Battle_of_the_Atlantic_(1939%E2%80%931945)#Outcomes).

Wrecks, both military and accidental, may serve as suitable or preferred habitat for a variety of suspension-feeding organisms and their associated fauna in an otherwise soft sediment environment. Colonisation of deep-sea wrecks is difficult to quantify. The *Titanic* has octocorals growing on the stem post and on chandeliers [113], but otherwise shows little evidence of colonisation. Two deep water wrecks, one in the eastern Atlantic (*Francois Vieljeux*) and one in the Mediterranean (SS *Persia*) are both somewhat surprisingly host to chemosynthetic fauna of the type normally found at cold seep sites [114], [115]. On the other hand, observation of the *Kumanovo* sunk in 1989 in 2500 m of water in the Gulf of Cadiz showed no colonisation at all (P Tyler pers. obs). In addition to ships, some 10,000 containers are lost overboard from ships each year, mainly as a result of storms (http://news.nationalgeographic.com/news/2001/06/0619_seacargo.html). Although some may float for weeks, many will sink to the seafloor taking their cargo with them. Impacts might be negative locally where the wreck/structure physically affects the seabed, but a hard substratum can increase local habitat heterogeneity providing substratum for certain species. However, the degradation of metals, paints and other material on board the wrecks can result in the release of toxic chemicals. A significant problem resides in ships containing munitions, as well as in discarded munitions, which, through the corrosive effect of seawater can release chemical pollutants. The impact of discarded or lost war material is difficult to assess. OSPAR (Commission for the Protection of the Marine Environment of the North-East Atlantic) has mapped chemical warfare components in the northeastern Atlantic [116] and a pioneer study (RED COD) has been conducted in a warfare material dumping site in the Adriatic [117]. The RED COD project showed that, although neither chemical warfare agents nor TNT were identified in tissues of the fish analysed, biomarker analyses indicated a higher stress level, higher arsenic and mercury content and gill DNA damage in the fish specimens from the dumping site compared to the non-impacted areas [117]. Taking into consideration the extent of war material dump sites worldwide [117] and the interconnectivity of oceans through hydrodynamic dispersal of particles, it is imperative that detailed studies be conducted assess local, regional and global impact of such material in the deep sea.

#### Carbon dioxide disposal

With increasing international interest in climate change and the recognised increase in CO_2_ levels in the atmosphere, methods proposed for the long term disposal of greenhouse gases include both sub-seabed disposal and surface seabed disposal [118]. The principle underlying sub-seabed disposal is that carbon dioxide (like methane) forms a solid crystalline structure at appropriate temperature and pressure conditions [119] and thus the injection of CO_2_ into suitable seabed structures (including past and ongoing oil and gas reservoirs) should cause the CO_2_ to form hydrates and hence act as a long term depository of excess CO_2_. The Sleipner gas field, a natural gas field in the North Sea, is already used as facility for carbon capture and storage (CCS) and is the world's first offshore CCS plant, operative since October 1996. Sleipner has stored about one million tonnes of CO_2_ a year at a depth of 800–1000 m below the seafloor. There has been no evidence of leakage so far and multinational and multidisciplinary research projects are underway to assess the CO_2_ state, to investigate any potential impacts and to predict the long-term destiny of the CO_2_ [120], [121]. This existing operation, and another in the Snøhvit gas field in the Barents Sea that stores 700,000 tonnes CO_2_ per year at depths of 320 m, are relatively small-scale when compared with proposed industrial scale CO_2_ disposal, which would store about 1000 times this amount.

A simpler and cheaper option that has been considered is the direct disposal of liquid CO_2_ onto the deep seabed, based on the principle that a gas hydrate will be generated on the seabed. Preliminary experiments have shown that surface disposal is feasible. However, small-scale experiments have shown that fish swimming into the CO_2_ plume are narcotised, although they recover as they drift out of the CO_2_ cloud [122]. Scavenging fish and amphipods appear able to detect and avoid toxic CO_2_ plumes released from hydrothermal vents at the seafloor [123], while some taxa have evolved tolerances at natural deep CO_2_ vents [124]. Experiments on the survival of meiobenthos exposed to small-scale patches of artificially emplaced liquid CO_2_ show that, immediately adjacent to the CO_2_, pH fell and meiofauna died, whereas at control sites about 40 m distant, pH was unaltered and no meiofauna died [125]–[128]. The experiments also assessed the survival of macrofauna and megafauna (i.e. gastropods, echinoids, holothurians, cephalopods and fish) during month-long exposure to elevated CO_2_ levels and concluded that disposal of human-generated CO_2_ in the deep sea will have variable, but generally negative effects on deep-sea ecosystems. Effects would be most pronounced near sites of CO_2_ release and depend on the volume of CO_2_ released [125]. Scaling up of these experiments to industrial levels would imply a potential major impact on benthic fauna at the disposal site. In particular, industrial scale CO_2_ disposal has the potential to create a “scavenger” sink, attracting and killing ever larger numbers of deep-sea scavengers drawn to an accumulation of dead biomass within the influence of the disposal plume [123]. An in situ experiment at more than 3000 m depth off Central California provided evidence that exposure to CO_2_-rich seawater is stressful for some deep-sea fauna such as harpacticoid copepods [129]. A frequently considered method of CO_2_ disposal with an indirect effect on the seabed is the use of iron fertilisation in areas of high nutrient low chlorophyll, to encourage phytoplankton growth, causing CO_2_ drawdown, with the subsequent sequestration of carbon via phytodetrital flux at depth [130]. Concerns have been raised about accompanying oxygen depletion [131]. Although iron fertilisation looks attractive in theory [132], [133] and is generating commercial interest, the amount of surface production sequestered is in the same order of magnitude as normal downward particle flux and thus relatively low [134]. Nonetheless, profound changes in ecosystem goods, services and values of the deep sea can be expected as a consequence of dumping iron into the ocean [135]. Ocean fertilisation by artificial upwelling has also been suggested to reduce the accumulation of anthropogenic carbon dioxide in the atmosphere. However, Oschlies et al. [136] recently greatly downplayed the benefits of this method. Their model suggests that most of the sequestered carbon (about 80%) would be stored on land because lower air temperatures caused by upwelling of cold waters would result in reduced respiration. Secondly, when artificial upwelling is stopped, the model predicts that surface temperatures and atmospheric CO_2_ would rise quickly for decades to centuries, reaching higher levels than those in a world that never experienced artificial upwelling [136].

### Resource exploitation

Whereas in the past the main threat to the deep sea was probably the disposal of waste solids and chemicals, as well as the cascading effects of overfishing in shallow water [137], new direct threats are appearing and increasing as a result of expanding technological capabilities that permit exploitation of biological, mineral and petrochemical resources.

#### Fishing: trawling and long lining

Technological development and market demand have both exacerbated the increasing exploitation of high-seas deepwater fisheries and the need to identify effective means of regulation to protect those fisheries and their environment [138]. Until the mid 1900s, trawling was generally restricted to the continental shelf at depths less than 200 m. However, from the late 1960s, the development of large, powerful factory trawlers enabled fishing activities in deep offshore waters. Because many major inshore stocks declined through the 1970s and regulations were introduced to reduce takes, fishing opportunities became limited in many continental shelf areas. The declaration of exclusive economic zones excluded a number of major fleets from their traditional fishing grounds. These factors led vessels to explore progressively deeper and more distant waters and new fishery resources [e.g. 28], [139], [140]. Fisheries on the upper continental slope and deep seamounts to depths of 1500 m expanded for species like pelagic armourhead (*Pseudopentaceros wheeleri*), orange roughy (*Hoplostethus atlanticus*), alfonsino (*Beryx splendens*), oreos (*Pseudocyttus maculatus*, *Allocyttus niger*) and grenadiers (*Coryphaenoides rupestris*), but almost none of these have proven sustainable [e.g. 28], [141], [142]. This deep-sea trawling has had an impact on fish populations down to 3100 m [143], as well as by-catch species [144]. Scientific knowledge of deep-sea fish populations has tended to lag behind fisheries development, and stock depletion often has occurred before the population dynamics of the exploited species were understood enough to be used to prevent stock collapse [e.g. 145]. Overfishing issues are particularly important in deep-sea species which are often long lived, with slow growth and delayed maturity [e.g. 146], making them poorly adapted to sustain heavy fishing pressure.

**Figure 4:**
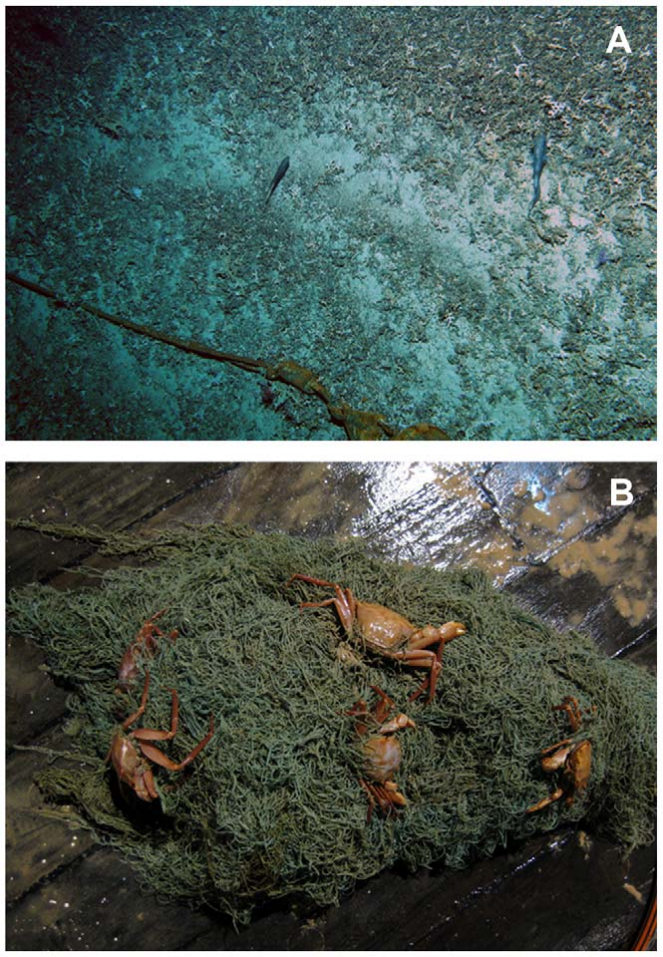
Fishing impact on deep-sea ecosystems. A, Part of a trawl lost on the seafloor and evidence of trawl disturbance and coral rubble on Zombie seamount, Chatham Rise, New Zealand EEZ (Photo courtesy of A. Rowden and M. Clark, NIWA); B, ghost fishing of *Geryon* crabs by a discarded/lost net recovered from 1200 m depth in the Western Mediterranean (Photo courtesy of E. Ramirez-Llodra, ICM-CSIC).

The effects of trawling on benthic habitat and communities can be severe in deeper waters, especially on the upper continental slope and seamounts [147]–[149]. On a global scale, most deep-sea bottom trawling happens on sedimentary slopes. In the OSPAR area (northeastern Atlantic), the spatial extent of bottom trawling is orders of magnitude greater than that of submarine cables, waste disposal and oil and gas exploitation [27]. Although the communities found at habitats such as seamounts, cold-water coral reefs and cold seeps may be more vulnerable than sediment-dwelling assemblages, the impacts of fishing on seamounts and cold seeps have rarely been assessed, with significant exceptions such as in New Zealand and Australian waters. Trawling effort can be intense, with hundreds, or even thousands, of tows repeatedly carried out on small seamounts or cold seeps [150], [151]. Heavy trawling can reduce the diversity and biomass of benthic invertebrates, especially framework-forming foundation species like cold-water corals [152], [153]. Recovery of cold-water assemblages from fishing disturbance occurs slowly, even after fishing has ceased for 5–10 years there have been no signs of faunal recovery [154], [155]. On the deep slope of the northwestern Mediterranean, trawling for the red shrimp *Aristeus antennatus* has taken place for the last six decades and is now reaching 900 m depth [156]. Recent biodiversity studies in this area suggest that there are significant differences in the community structure of fished and non-fished areas, with a decrease in sessile and fragile species such as corals, sponges and echinoderms on the impacted fished seafloor [24], [157]. Hence, as in shallow water, bottom trawling has a large impact on areas of deep slope and is greater still in rocky areas or seamounts where coral is frequently found at depths of about 1000 m (Figure 4A). Trawling over the corals breaks up the reef-like structures that may take decades or centuries to re-establish [158]. There is recent evidence that some gorgonian octocorals taken as by-catch exceed 4000 years in age [159]. Furthermore, studies in the northwestern Mediterranean have shown that intense repetitive trawling on the slope and on canyon flanks can create significant disturbance to the sediment, causing sediment gravity flows [160], [161]. The sediment eroded by fishing trawls between 400 and 700 m depth was channelled by gullies to the canyon axis and recorded down to 1200 m depth. This suggests that intense trawling in certain regions needs to be taken into consideration for canyon sediment dynamics and that the gravity flows generated can have major consequences (e.g. suffocation of cold-water corals) far from the trawled area [160]. A further issue with industrial fishing is the presence of lost or discarded nets on the seafloor, which are responsible for ghost fishing affecting the benthic and benthopelagic fauna passively for years (Figure 4B).

Longline fisheries have also worked progressively deeper in recent decades. They are used particularly in areas that are not fishable by trawl because of rocky outcrops, rugged terrain such as seamounts and canyons, or fisheries regulations. Fishing profitability is often considered higher near coral concentrations than elsewhere [162]. Longline operations in the Southern Ocean for toothfish (*Dissostichus eleginoides*, *D. mawsoni*) can extend to below 2000 m depth. Although long-line fishing may have less direct impact than bottom trawling, the line weights and the line itself can cause damage to benthic fauna, especially erect sponges and corals [e.g. 28]. Impacts of longlining have not been well quantified in any area but, like the effects of bottom trawling, these will depend on the intensity of fishing and the spatial distribution of fishing effort in relation to sensitive habitats, such as coral-rich areas. Lines suspended off the bottom or vertical lines will have lesser impact on benthic fauna than bottom-set gear. Fish often aggregate on continental slopes near carbonate hard-grounds associated with methane seepage, or near corals that settle on these carbonates. Recently discovered seeps off Chile and New Zealand were first located by fishermen who recovered chemosynthetically-driven species in nets and on lines, and subsequent exploration of these sites suggests extensive damage occurred before these seep habitats were even known to exist [151], [163].

As vessels become more technologically advanced and electronic monitoring of fishing gear becomes more accurate and reliable, fishing can occur at ever increasing depths. It is conceivable, in principle, that many deep-sea habitats and their communities could be affected by commercial fishing in the coming years. However, as most target species are only distributed on upper slopes of continents, ridges and seamounts, fishing deeper is unlikely to be very attractive. In addition, conditions in the early days of industrialised deepwater fisheries (1970s–1990s), when catches increased and cases of severe depletion were recorded, different from sharply from the current situation. After 2005, all statistics show declining trends in landings [28], [140] which may be attributed to decreasing abundance of resources [e.g. 144] and/or decreasing fishing effort. Fishing activity depends primarily on economic incentive. Reduction in subsidies in many countries, rising fuel costs, and recent introduction of stricter regulations mean that industry must perceive the prospects of deep-water fishing as highly favourable before engaging in it. Limiting factors include the low quality of some deep-sea fish caused by the high water content of their muscles, the taste of some fish, for example from hydrothermal vents, and low profitability in the case of the deeper regions such as abyssal plains and trenches [140]. Offshore seamount fisheries are, even now, focusing on high value species that can be taken in small quantities [152].

Over the past decade, management and protection measures have been developed by coastal states and regional fisheries management organizations, recently in response to UN General Assembly resolutions. In addition to traditional quota management and licensing systems, an increasing number of seamounts, upper slope and ridge areas are being closed to fishing operations around the world [140], [164]. Guidelines have been prepared to help improve the sustainability of deep-sea fisheries and reduce the environmental issues associated with fishing [165], [166]. For example, in the Mediterranean, a coordinated effort between scientists, the International Union for Conservation of Nature (IUCN) and World Wildlife Fund (WWF) [167] resulted in 2005 in the legal ban by the General Fisheries Commission for the Mediterranean of bottom trawling below 1000 m depth and of driftnet fishing for the whole Mediterranean, applying the precautionary approach. Off New Zealand, almost one third of the exclusive economic zone (EEZ) is closed to bottom trawling as “Benthic Protected Areas” (the majority of the seafloor area being deeper than current trawling practices allow) [168]. Other large closures or restrictions have recently occurred in the deep sea off Alaska, Hawaii, the Azores, the North Atlantic Ocean [169] and the North Pacific. Regional Fisheries Management Organisations (RFMOs) are becoming more active in regulating deepwater fishing activity on the high seas and make use of many different instruments, including the precautionary closures of large oceanic areas. The high seas are still lacking a fully coordinated approach or network of conservation areas [164]. Market measures aimed to combat illegal and unreported fishing have been introduced in several regions. In Europe, the Oceans 2012 initiative is designed to ensure that the 2012 reform of the EU Common Fisheries Policy includes tools to stop overfishing and ends destructive fishing practices, as well as ensuring an equitable use of healthy fish stocks (www.ocean2012.eu).

Recently, new fishing rules aimed at protecting vulnerable marine ecosystems (primarily benthic communities) in international waters have been implemented by several RFMOs (e.g. General Fisheries Commission for the Mediterranean (www.gfcm.org), Northeast Atlantic Fisheries Commission (www.neafc.org), Northwest Atlantic Fisheries Organisation (www.nafo.int), South-East Atlantic Fisheries Organisation (www.seafo.org), Commission for the Conservation of Antarctic Marine Living Resources (www.ccamlr.org) and South Pacific Regional Fisheries Management Organisation (www.southpacificrfmo.org) [170], [171]. This is a process in progress and the effectiveness of the approaches cannot yet be fully assessed.

#### Mining

Three forms of deep-sea mineral resources have been considered thus far for commercial exploitation: manganese nodule mining on abyssal plains [172], cobalt-rich crusts on seamounts [173] and massive polymetallic sulphide deposits at sites of hydrothermal venting [174].

**Figure 5:**
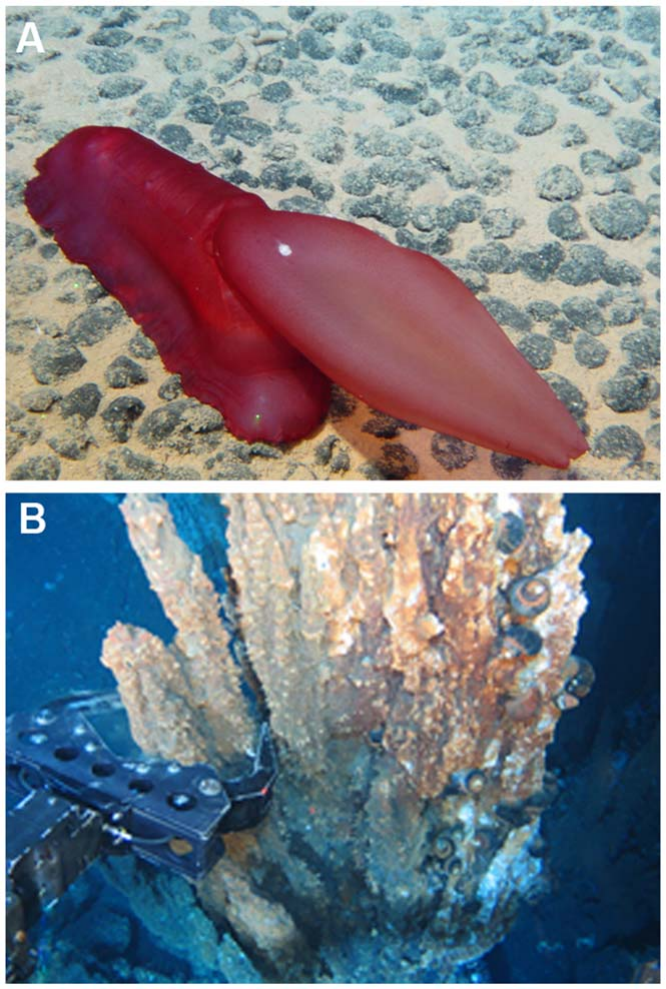
Exploitation of deep-sea mineral resources. A, the holothurian *Psychropotes semperiana* over manganese nodules on the Kaplan abyssal plain in the Pacific Ocean (Photo courtesy of Ifremer - Nautile/Nodinaut, 2004); B, sampling a vent chimney off Papua New Guinea during the environmental assessment conducted by Nautilus Minerals before exploitation of massive sulphides (Photo courtesy of Nautilus Minerals).

Manganese nodules are found in many areas of the abyssal seafloor beneath regions of low to moderate primary productivity (Figure 5A). Manganese nodules provide a potentially enormous source of copper, nickel and cobalt, metals now in high demand in the rapidly growing economies of developing countries. Manganese nodule mining may not occur for another 10–15 years, but it could ultimately be the largest scale human activity to impact the deep-sea floor directly. At present, nine contractors have registered nodule-mining exploration claims with the International Seabed Authority (ISA) in the central Pacific and Indian oceans, with each claim area encompassing 75,000 km^2^ [175]. Most of the claim areas fall in the Clarion-Clipperton Zone of the Pacific, between 8–17°N and 120–153°W. A single mining operation is projected to remove nodules and near-surface sediments from 300–700 km^2^ of seafloor per year, yielding near total faunal mortality in the area directly mined. Re-deposition of sediments suspended by mining activities will disturb seafloor communities over an area perhaps two to five times greater [175]. Thus, over a 15-year period, a single mining operation could severely damage abyssal communities over an area of 50,000 km^2^ and three mining operations might severally disturb a seafloor area half the size of Germany.

Nodule mining will have a variety of impacts at the deep-sea floor. The most obvious direct impact will be removal of the nodules themselves, which will require millions of years to re-grow [176], [177]. Manganese nodules provide the only hard substratum over much of the abyssal seafloor, so mining will remove permanently a major habitat type, causing local extinction of the nodule fauna, which is substantially different from the sediment-dwelling benthos [178]–[181]. Nodule-mining activities will also remove roughly the top 5 cm of sediment, potentially re-suspending this material into the water column [182], [183]. The nodule-mining head will immediately kill most of the fauna directly in its path and communities in the general mining vicinity will be buried under varying depths of sediment [182]–[186]. Abyssal nodule habitats are among the most stable on Earth and are dominated by very small, fragile deposit feeders exploiting a thin veneer of organic matter near the sediment-water interface. Thus, the mechanical and burial disturbances resulting from commercial-scale nodule mining are likely to be devastating [35], [184]. A limited number of *in situ* experiments have been conducted to evaluate the sensitivity and recovery times of abyssal benthic communities to simulated mining disturbance. Although the experimental disturbances created were substantially smaller in intensity and many orders of magnitude smaller in spatial scale than is expected from commercial mining, they provide important insights into the sensitivity and minimum recovery times of abyssal nodule communities following mining [reviewed in 25], [35], [186]. It is clear from these experiments, that abyssal communities will be dramatically disturbed by less than 1 cm of sediment redeposition resulting from mining, and that full community recovery from major mining disturbance will take more than seven years and possibly even centuries. Unfortunately, these experiments do not allow prediction of the likelihood of species extinctions from nodule mining because the typical geographical ranges of species living within the nodule regions are unknown. Species turnover does occur across the nodule region, especially with latitudinal changes in overlying productivity [175], so large-scale mining activities have real potential to yield species extinction. Nonetheless, it is clear that effective management of the environmental impacts of commercial scale mining requires substantially more information concerning species ranges, sensitivity to sediment burial and the scale dependence of recolonisation processes in abyssal seafloor communities. A workshop at Manoa, Hawaii, in October 2007 [187] produced a rationale and recommendations for the establishment of “preservation reference areas” in the Clarion-Clipperton Zone, where nodule mining would be prohibited in order to leave the natural environment intact.

Cobalt-rich ferromanganese crusts occur on seamounts, ridges and plateaus where crust minerals precipitate out onto rocky surfaces that currents sweep clean of sediments over long periods [188]. These crusts occur universally on exposed rocks throughout the oceans, but form thick pavements (up to 250 mm thick) primarily on large seamounts and guyots in the western and central Pacific Ocean [188]–[191]. The chemical composition of the crusts can be high in manganese and iron, and the exploitable minerals include cobalt, copper and platinum. Such crusts could provide up to 20% of the global cobalt demand [192]. However, exploitation has not yet proven cost-effective [66], [173]. Little research has been conducted on the influence of the chemical composition of a hard substratum on seabed communities. The biological communities associated with the particular chemical environment at, and surrounding, active hydrothermal vents have been extensively studied in recent decades [e.g. 193], but much less is known about the fauna of cobalt-rich crusts on seamounts [194]. Recent work conducted for the International Seabed Authority (ISA) compared the fauna observed in submersible dives on cobalt-rich and non-cobalt-rich crust seamounts off Hawaii [195]. The study found fauna were similar on both types of seamount, although more detailed studies are currently underway.

More recently, there has been considerable interest in metal rich deposits of seafloor massive polymetallic sulphides [174], [196]. Massive sulphide deposits are laid down as a result of hydrothermal activity and can be many metres deep, weighing from several thousand to 100 million tonnes and containing high concentrations of zinc, copper, lead, cadmium, gold and silver [174]. Most precious metals are being evaluated as potential future resources under both national (EEZ) and international (UNCLOS and ISA) regimes [197]. The mining industry is at an advanced exploration stage. Two main companies have developed exploratory studies and environmental impact assessments: Nautilus Minerals Inc. and Neptune Minerals (Figure 5B). Both companies are working in deep waters of the exclusive economic zone of individual nation states. Nautilus Minerals is active in the Manus Basin, in Papua New Guinea waters, at 1500 m depth, but also has licences for areas in New Zealand, Fiji, Tonga and the Solomon Islands. Neptune Minerals have focused its exploratory activities in the New Zealand area, with exploration licenses also in Papua New Guinea, Vanuatu and Micronesia. The mining industries focus their attention on presumably inactive vent sites, where mining would be less hazardous to humans and the ecological impacts to hydrothermal vent communities would be smaller. However, it has been shown that these inactive vents support chemoautotrophically-based food webs [198] and they are located within active vent fields. Although detailed environmental surveys of the region have been conducted, preparatory to obtaining approval for mining, the real nature of the impact is still not well understood.

Potential impacts from mining massive sulphides include the physical destruction of the mined vent sites and their fauna, production of sediment plumes affecting filter feeders, changes in hydrothermal circulation at the active sites, wastewater and potential chemical pollution from equipment failure. Following the environmental assessments, measures are under consideration to minimise these potential impacts. For example, the sediment plume will be minimised by bringing all mined material up to the surface support vessel, where it will be filtered before the water is discarded back into the ocean at depth. However, other impacts are inevitable, such as habitat and fauna destruction at the mining site. This site disturbance is particularly important in a habitat such as inactive vents where little is known about their faunal communities and the interaction of the fauna with that of nearby active sites [198]. Levin et al. [199] conducted a comparative study of the macrobenthos community within sediments of active and inactive vent sites in the Manus Basin (southwestern Pacific) where commercial mining of massive sulphides is planned and in Middle Valley (northeastern Pacific). The active sites showed a higher abundance and density of macrofauna and lower diversity than the inactive sites and there were significant differences in community structure between Manus Basin and Middle Valley, as well as significant heterogeneity within the region [199]. The authors highlight the need to understand species endemicity, distribution and reproductive patterns for effective management, as the potential loss of rare species or species with low colonisation potential could be a significant risk.

The increased likelihood of mining at hydrothermal vents has led to recent activity among different working groups aimed at developing guidelines for protection and identifying where knowledge is needed to ensure effective environmental management of mining. The Census ChEss Programme and InterRidge programme Seafloor Mineralization working group held a workshop and a public colloquium on massive sulphide mining (Woods Hole Oceanographic Institution, 2009) and produced a set of questions and recommendations for research [196], [200], [201]. A recent workshop sponsored by the deep-water Census of Marine Life projects ChEss, COMARGE, CeDAMar and SYNDEEP, as well as InterRidge, the International Seabed Authority (ISA), US Minerals Management Service (MMS), U.S. National Oceanic and Atmospheric Administration (NOAA), NOAA's National Marine Sanctuaries and the French Centre de Recherche et d'Enseignement sur les Systèmes Cotiers (CRESCO), identified mining as a pending threat to hydrothermal vents and developed marine protected area (MPA) guidelines for both hydrothermal vents and cold seeps [202].

#### Oil and gas exploration and extraction

**Figure 6:**
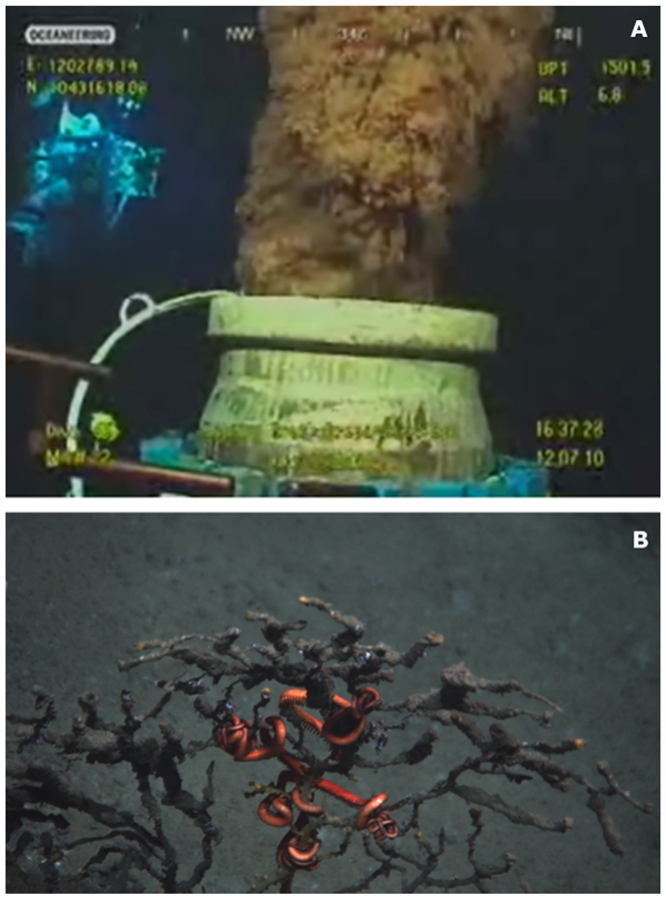
Deepwater Horizon oil slick in the Gulf of Mexico, 2010. A, photo of the oil being discharged in the water column (open source image); B, a coral in the deep Gulf of Mexico, with attached ophiuroid, covered with oil (Photo courtesy of Lophelia II 2010, NOAA OER and BOEMRE).

In the last 20 years, oil and gas exploration has extended into deeper water with oil wells being drilled in 3000 m of water [203], with an increased risk of drilling muds and accidental oil spillage affecting deep-sea habitats. The main effects of oil exploration and exploitation are on the continental margin habitats, including sedimentary slopes, seeps, vents (e.g. oil from early diagenetic processes as in the Guaymas Basin), oxygen-minimum zones and possibly in areas where there are corals.. A study of the effects of oil and gas exploration and exploitation in the Gulf of Mexico showed that drilling muds were deposited in the near-field areas, causing elevated total organic carbon, anoxic conditions and patchy zones of disturbed benthic communities [204]. The oil industry generally has shown considerable environmental responsibility in its exploration of the deep sea and most contamination is largely the result of accidental discharge. However, in April 2010, there was a major accident in the Gulf of Mexico where safety valves exploded and oil mixed with gas was released from the Deepwater Horizon well directly into the deep sea (http://www.bp.com/gulfofmexicoresponse). The explosion caused the released of about 5 million barrels (780×10^3^ m^3^) of crude oil into the water (Figure 6A). The well was finally sealed on 19^th^ September 2010. A significant reduction of dissolved oxygen in the water column and effects of chemical dispersants that had been added to the spill (E. Escobar, pers. obs.: SIGSBEE.13 cruise Aug. 19–Sept. 2, 2010) were observed a month after the well closure. The oil reduction was accompanied by the presence of significant chromophoric dissolved organic matter fluorescence anomalies [205] below 1500 m depth. The lower dissolved oxygen values have been interpreted as the weathering of the oil and biodegradation of hydrocarbons in the deep water by bacteria in its metabolic pathway for hydrocarbon degradation. However, a study of the plume of oil that persisted for months at 1100 m depth showed that the monoaromatic petroleum hydrocarbon input to the plume was more than double the total amount produced by all natural Gulf of Mexico seeps and no biodegradation was observed [206]. The immediate impact of the spill on the deep-sea ecosystem was mostly local in the Gulf of Mexico. However, a research cruise was organised in December 2010 to analyse the seep communities in the area with the *Alvin* submersible (C. Fisher, pers. comm.). First observations showed colonies of the coral *Madrepora* as well as soft coral, covered with oil at 1400 m depth. The corals were recently dead or dying and the symbiont ophiuroids often attached to them were also affected (http://www.science.psu.edu/news-and-events/2010-news/Fisher11-2010) (Figure 6B). A series of facts (proximity of the site to the oil spill, depth, clear evidence of recent impact, and three decades of background data in this area) suggest that the impact observed caused exposure of the biological community to oil, dispersant, extremely depleted oxygen, or some combination of these effects of the spill (C. Fisher, pers. com.). The occurrence of natural hydrocarbon seepage in the region, which fuels fragile methane seep ecosystems locally, raises questions about the ability of resident microbes and fauna to cope with excessive amounts of oil. The oxygen levels could decrease in the deep water if a significant fraction of oil remains in the subsurface and the rate of dispersion of the oil is low. However, mid depths in the Gulf of Mexico experience a natural oxygen minimum and it remains uncertain whether exacerbated large-scale hypoxia could result as a consequence of microbially-mediated oxidation of the oil [205]. The 2010 Deepwater Horizon leak in the Gulf of Mexico has proven to be the largest accidental oil spill into the ocean in world history, surpassed only by the intentional 1991 Gulf War spill in Kuwait [207]. Modelling of deep-water releases of gas and oil from deep-water blowouts is essential in risk assessments to predict plume behaviour, size distribution of oil droplets and the fate of water-soluble oil components from dispersed oil droplets. This information is required to understand the potential impact of the blowout to the deep-water fauna and surface waters [208], [209].

As well as the threat of accidental oil and gas discharges, the purposeful disposal of obsolete structures (e.g. buoys, rigs, mooring blocks and cables) is of some concern. In the mid 1990s, Shell proposed the disposal at sea of a large metal structure, the oil storage buoy Brent Spar. However, the political controversy this created ensured that such structures may well never be disposed of in the deep sea [210]. There is also evidence that, as oil exploration moves into deeper water, cold-water corals colonise the legs of oil rigs [211], [212]. In relatively shallower waters (100–150 m depth), the recovery for scrapping of steel structures from the Frigg oil field in the North Sea showed considerable coral growth after sitting in a presumed “coral-free” area since the 1970s (Bergstad, pers. com.).

A currently untapped potential energy resource on the mid continental margin is the massive reservoir of gas hydrates (frozen methane). Methane hydrate is a solid form in which water molecules trap methane without binding to them. The U.S. Geological Survey estimates that 200,000 trillion cubic feet of methane may be present in the United Sates and its margins. This is about 2000 times the amount of energy the United States consumes in a year. The gas hydrate province on the eastern margin of New Zealand's North Island covers an area of 50,000 km^2^, with a total estimate of 23,000 km^3^ of recoverable gas including up to 12.5 km^3^ concentrated in “sweet spots” suitable for commercial production [213]. Global estimates of methane hydrate volumes are less certain, but are considered to be 10 times the recoverable natural gas supply. Over time, estimated global reserves have decreased from 530×10^15^ g of carbon (530,000 Gt C) [214] to a minimal possible estimate of 0.1×10^15^ g of carbon (100 Gt C) [215]. Widely cited current estimates still range from 500 to 63,400 Gt C, distributed in 90 locations along the continental margin. Of the empirical estimates, arguably the “consensus” value of 10,000 Gt C [216] obtained independently by Kvenvolden [217] and MacDonald [218] is still the most widely quoted. It remains unclear whether methane can be safely or economically extracted from gas hydrate resources in a useable form. Pilot plants have been constructed to test extraction technologies, mainly at very high latitudes (e.g., Alaska North slope), but the technology remains in its infancy. Most gas hydrates are buried beneath a thick sediment cap on the sea floor below 250 m. In places where gas hydrates intercept the sediment surface, or where dissociation of methane occurs, methane seep ecosystems are well developed. Should mass extraction of gas hydrates become a reality, many methane seeps might become subject to disturbance more significant than that of oil and gas extraction, unless protection is put in place, as in the Gulf of Mexico [219]. Physical disruption of sediments and intensified currents, release of high salinity and anoxic water during production process, loss of energy sources fuelling microbes at the base of seep food chains, altered habitat structure and introduced substrate are possible ecological effects of hydrate mining. Because seep habitats are often small and patchy in nature, a better understanding of seep meta-population and meta-community dynamics is needed to assess the consequence of localized disturbance. Removal of gas hydrate presents larger-scale geohazards and might trigger mass instabilities. Since natural fluxes of methane from the deep-sea floor are so poorly known, it will be difficult to estimate the further atmospheric and climate effects of active methane extraction and release from gas hydrates.

#### Underwater cables

The laying of underwater telegraph cables came early in our understanding of the deep sea. HMS *Cyclops* in 1855 was used to determine the depth profile between the UK and Newfoundland for the laying of the first transatlantic cable. The first effort in 1857 failed when the cable-dispensing machinery became disabled and cut the wire, but the cable was finally successfully connected in 1858 [81]. In subsequent years, cables were laid in many parts of the global oceans. It was the recovery of a broken cable from 2180 m between Sardinia and Bona, encrusted with the coral *Caryophyllia* that demonstrated the viability of life at lower bathyal depths. In the northeastern Atlantic, a maximum spatial extent of submarine cables in the OSPAR northeastern Atlantic area has been estimated to range between 5 and 10 km^2^, although this is most likely an underesftimate as it does not take into account the effects of plough burial [27]. Pipelines offer a similar scenario, although they tend to be physically bigger than cables. We predict minimal impact of underwater cables (Tables S2 and S3).

#### Scientific activity

Since the onset of dedicated deep-sea research, sampling methodologies have evolved continuously and the number of research expeditions investigating the deep seafloor has increased regularly. Trawls, dredges, grabs, box cores and other sampling apparatus used to collect animals have an impact on the deep-sea habitat that is comparable in type, but not duration, spatial scale or magnitude of the disturbance to that caused by industrial removal of seafloor resources. The modern use of submersibles and ROVs adds a new type of impact – light – to the more established physical impact of sample collection and discarded material such as ballast weights and site markers. At Atlantic hydrothermal vents, there was concern [220] about the effect of submersible lights on the sensitivity and integrity of the dorsal photoreceptor of the vent shrimp *Rimicaris exoculata*. Ultrastructural changes in the dorsal organ of shrimp exposed to submersible lights was demonstrated [221], but no detectable changes to the shrimp population abundance at a shrimp-dominated site (TAG) on the Mid-Atlantic Ridge have been observed during the more than 20 years since submersible observations first began [222]. The inference is that, although a percentage of the shrimp population is likely to have been blinded during episodic visits by submersibles, the role of light detection in the survival of shrimp is of less importance than other sensory modalities (e.g. chemosensory) [223]. During a recent expedition, scientists assessed the effect of research using the French submersible *Nautile* at hydrothermal vents, evaluating the effect of chimney collection, coring and discarded material on the seafloor. The conclusion was that most of the impact occurs at local scale and is thought to be minor, although two aspects need further evaluation: the effect of material left behind at sites after experiments are conducted (e.g. plastic, ropes) and the effect of iron and other metals from submersible ballast weights on the habitat and communities at study sites (E. Escobar, pers. com.). Taking into account the concern for damage caused by repeated sampling for scientific purposes, the international science community led by InterRidge and the Census of Marine Life programme ChEss coordinated the writing of a Code of Conduct for best practice when sampling deep-water hydrothermal vents [224]. A recent world-wide survey of the use of the Code has been carried out showing that although most consulted deep-sea scientists were aware and supportive of the code, there was a lack of information and confidence of the respect other scientists have for the code [225]. The authors of the survey suggest that protection of specific vents is necessary in parallel with the code to ensure the sustainable use of hydrothermal vent ecosystems for all stakeholders.

#### Bioprospecting

The high biodiversity in the deep sea may make this ecosystem a valuable resource for biological and genetic materials of potential commercial value, the recovery of which is usually referred to as bioprospecting. In the deep sea, such bioprospecting is in its infancy with reports generally suggesting only where suitable materials could be obtained [226]. To date, research and product development have centred mainly on the development of novel enzymes for use in a range of industrial and manufacturing processes, and DNA polymerases for use in research and diagnosis. More recently, some research has been directed toward possible pharmaceutical and therapeutic applications such as antifungals, anti-cancer products and skin protection products. Investigations are also underway regarding the possibility of making artificial blood from the haemoglobin found in the blood of vent tubeworms [227]. The main difficulty with deep-sea bioprospecting is the technology required to collect and preserve animal tissues in a way biological materials can be extracted and exploited. In the deep sea, the extreme conditions of pressure, temperature and chemical concentrations found at hydrothermal vents lead to specific physiological adaptations that can be useful for pharmaceutical and technological industries, whereas seamounts have a diverse macro- and megafauna concentrated in a restricted area that increases the potential of finding species with particular characteristics attractive to industry [226]. However, bioprospecting on and under the high seas raises a variety of legal and ethical issues. The patenting of a whole genome (*Methanococcus jannaschii* from the deep seabed) will have different implications to that of an endangered species, an extract or a chemical compound. The present international legal framework, encompassing the United Nations Convention on the Law of the Sea (UNCLOS) and the Convention on Biological Diversity (CBD), does not adequately address the conservation of, access to, and benefit-sharing related to deep seabed bioresources [226].

### Ocean acidification and climate change

Although climate change has taken place in the past, the time scale has been geological. Drastic climate changes have occurred after catastrophic events that led to mass extinctions on Earth that could not be overcome by evolutionary adaptation. For the first time in Earth's history, however, climate change is being driven by human forcing and proceeding at a pace that may outstrip evolutionary change. Climate change is affecting the marine environment and the deep sea is not immune from the consequences [130]. In the deep-sea ecosystem, climate change implies a series of significant processes such as a rise in CO_2_ levels and ocean acidification, temperature change, expansion of hypoxic zones, destabilization of the slopes and gas hydrates and changes in productivity regimes. In contrast to the previous examples of human impact on the deep sea, where there are measurable data, much of our understanding of the impact of climate change is speculative, in part because there are only a few sites with the long-term baseline data needed to document biological changes [reviewed in 228].

#### Ocean acidification

The atmospheric partial pressure of carbon dioxide is currently the highest experienced on Earth for the last 20 million years, and is estimated by 2100 to be double that of pre-industrial times [229]. Closely associated with increased atmospheric CO_2_ and global warming is decreased pH in the water column. The ocean is a natural sink for CO_2_ but has also absorbed half the anthropogenic CO_2_ in the atmosphere, causing acidification. At present the pH of seawater is 0.1 units lower than that in the early 1900s, and by 2100 it is estimated to decrease by 0.4 to 0.5 units [230]–[232]. One of the effects is a lowered calcium carbonate saturation state of colder waters. This change can have a profound impact on calcifying fauna. Aragonite, high magnesium calcite and calcite are the main calcium carbonate crystals made by these organisms and, because high magnesium calcite and aragonite are more soluble than calcite, the species that use these compounds – such as scleractinian corals and echinoderms – are more vulnerable and will be the first to be affected [233]–[236]. Deep-water corals are one of the most important taxa to be affected, both because of their contribution to deep-water diversity and because of their structural role in providing habitat to a variety of other species [237]. The distribution of cold-water corals already reflects the acidic conditions in the North Pacific [238] but, in the long term, the entire ecosystem could be threatened by acidification. The calcium carbonate compensation depth (CCCD) varies with ocean, being the shallowest in Antarctic waters, but as CO_2_ builds up the CCCD will move toward the surface. Echinoderms, which have skeletons of high magnesium calcite, the most soluble form of carbonate, are likely to be among the taxa most affected by acidification in deep water. Their relative paucity in low-pH oxygen minimum zone (OMZ) waters [239], and the high susceptibility of their larvae to developmental abnormalities at low pH [240] support this conjecture. The shallowing of the CCCD has been predicted to leave the majority of deep-sea stony corals in water unsuitable for obtaining aragonite for building their skeletons [239]. Habitat suitable for stony corals is predicted, under future climate scenarios, to be particularly reduced in the North Atlantic [241]. Molluscs, which often have aragonitic shells, will also be susceptible to damage, while foraminifera, with calcitic tests may be least affected. Early life stages of calcifying species may be more susceptible to acidification effects than adults [240], [242]. A decline in the numbers of some species will also have a secondary effect on fish stocks in some circumstances (e.g. pteropods on fish stocks).

#### Climate warming and hypoxia

Ocean surface temperaftures are predicted to rise between 1.4° C and 5.8° C in the next 100 years [231]. Variations in surface temperature will have several inter-related effects with potentially significant impacts on deep benthic communities. Increasing surface temperatures may affect the formation of cold oxygenated deep water, modifying global ocean circulation and the dissolved oxygen availability in deep-water masses, increasing the existing natural OMZs. Although not all scientists agree with these predictions [243], the ultimate effect of a significant temperature rise might be the cessation (or minimization) of the deep thermohaline circulation that ensures the oxygenation of the deep sea. There is evidence of decadal changes of abyssal temperature in the Pacific Ocean [244], the Caribbean [245] and Antarctic Bottom Water [246]. Throughout the oceans, warming decreases oxygen solubility and increases stratification of seawater (enhanced by ice melt), which reduces vertical mixing and oxygen inputs. The stratification of the world's oceans is increasing by about 800,000 km^2^ per year, with the greatest change in the North Pacific [247]. Given the multiple mechanisms at play, it is not surprising that reduced oxygenation of the ocean's interior has already been documented [reviewed in 248]. Current models predict an oxygen decline of 1% to 7% in the next 100 years [248] with an expansion of pelagic and benthic OMZs [249]. Documented oxygen declines appear to be greatest between 200–700 m in the subtropical and tropical oceans globally [250], and in the northeast Pacific Ocean [251]. Off southern California, oxygen has declined by 20% to 30% at 200–300 m over the last few decades, and the hypoxic boundary has shoaled by nearly 100 m [252]. This change has been attributed to increased stratification [252] and to strengthening of the California Undercurrent, which transports low-oxygen subtropical water northward [253].

Expansion of OMZs will undoubtedly alter the composition, diversity and functional properties of bathyal ecosystems. For the majority of pelagic species that are not tolerant of hypoxia, a shoaling of OMZs causes vertical habitat compression, increased species encounter rates and possibly reduced vertical migratory range. Billfish in the tropical Pacific [254] and Atlantic [255] experience this compression; they are larger as prey become more concentrated, but they are also much more susceptible to fishing mortality. However, species such as the Humboldt (Jumbo) squid (*Dosidicus gigas*) with affinities to low-oxygen waters will expand their ranges vertically and horizontally. *Dosidicus gigas* has moved northward in the eastern Pacific and is now routinely found off Oregon, Washington and Alaska [256]. Because jellyfish are relatively tolerant of hypoxia and can store oxygen in their mesoglea, the jelly plankton may also benefit in a lower-oxygen ocean. Benthic communities within core regions of the OMZ are typically composed largely of nematodes, annelids and molluscs, with few crustaceans and echinoderms [239], and bacterial mats may cover the seabed in patches [257]. OMZs also exhibit low pH, contributing to stress and reduced densities of calcifiers [258]. Faunal assemblages exhibit low density, low diversity, and sometimes small body size [239], [259]. Likely functional consequences of expanding OMZs include increased roles for chemosynthesis in trophic pathways [239], a shift in carbon processing from metazoans to protozoans [260], and reduced rates of bioturbation and carbon burial [261], [262]. However, high faunal densities of a limited number of species can occur just above their threshold oxygen tolerance levels (in lower OMZ transition zones), where food is abundant and predator densities are reduced [239], [263].

Another possible concomitant effect of warming could be the release of methane from gas hydrates buried beneath the seafloor. The methane reservoir in gas hydrates in the seabed and in permafrost is so large that if 10% of the methane were released, its effect on the Earth's radiation budget would be equivalent to a tenfold increase in CO_2_ [264]. Increases in deep-water temperature of only 3° C could destabilise the delicate structure of methane hydrate deposits that occur on the continental slope. This destabilisation would release methane that may reach the atmosphere with a positive feedback to global climate and altered distribution of cold seep ecosystems [217]. The mechanisms in which hydrates may be destabilized on the continental margin and slope, the rate and pathways by which methane gas released from hydrates on the sea floor might be transferred to the atmosphere, are still matters of debate. Originally the potential for catastrophic methane release over human lifetimes (termed the clathrate gun hypothesis) was considered possible [265], but now it is argued that massive methane release from deep water could only occur over thousands of years or millennia [266]–[268]. However, the possibility of present large-scale methane releases caused by climate change [268], [269] through mechanisms triggered by deep-water warming [270], [271], mass wasting on continental slopes [272] and slumping of the sea floor with release of solid hydrates is still debated as a way of transfer for the seafloor gas from gas-hydrates to the atmosphere [273]. This process of gaseous methane plumes rising from the seafloor and reaching the atmosphere was studied experimentally by breaking a solid hydrate and following the gas plume to the surface [274]. Methane in the Arctic may be the most vulnerable to release by warming [264]. Such releases of methane caused by atmospheric warning have been linked to past extinction events such as occurred in the Palaeocene-Eocene Thermal Maximum (56 million years ago) and during the Permian-Triassic transition (251 my ago) [275].

#### Productivity changes

Ocean stratification decreases nutrient availability and surface productivity, consequently diminishing the flux to the deep-sea bed. Because most deep-sea fauna are heterotrophic, this change would have significant effects on the trophic structure of deep-sea communities. An increase in CO_2_ levels from the present day 384 ppm to 540 ppm will increase surface temperature, reduce surface productivity and cause a transfer from diatoms and large zooplankton to picoplankton and microzooplankton, with a concomitant decrease in the flux from surface production to deep waters [130]. This flux reduction leads to a lower deep-sea benthic biomass, reduced abundance and smaller body size as well as a decrease in sediment community oxygen consumption. The present and potential future impact is mostly unknown, but recent studies have related climate-induced surface productivity change to community change in the deep-sea benthos [276]. For example, in the abyssal northeastern Atlantic, a significant community structure change with a significant increase of the elasipodid holothurian *Amperima rosea* was observed in the mid 1990s [277]. This community change was related to changes in the reproductive output of *A. rosea* linked to variations in the quantity and quality of phytodetritus input [278], [279]. In the Eastern Mediterranean, a major climate anomaly took place in the early 1990s, with a sudden decrease of 0.4°C in water temperature and changes that modified the physico-chemical conditions of the system, including changes in organic matter input to the seafloor [280]. This led to changes in nematode biodiversity, community structure and ecosystem function [281], [282], and the observed increase in food availability resulted in an increase in metazoan abundance [283], [284].

#### Large episodic events

Climate change may also affect the periodicity and intensity of episodic events such as dense shelf water cascading [110]. The effects of these climate-driven oceanographic mesoscale processes on the ecosystem are poorly understood and are currently under investigation. However, pioneer studies in the northwestern Mediterranean have shown a link between cascading events and the significant decrease of the commercial rose shrimp *Aristeus antennatus* from the fishing grounds. This reduction in the shrimp population produces a temporary fishery collapse, but three to five years after the event an increase in the abundance of shrimp juveniles is observed and landings increase again to normal levels [111]. The authors suggest that this process is responsible for the long-term maintenance of the shrimp population, mitigating the effects of over-exploitation.

#### Acidification and climate change summary

Effects of acidification, deoxygenation, warming and localised methane release on deep-sea ecosystems remain key research agenda items. Changes in pCO_2_, temperature, oxygenation and methane will not occur in isolation, but will co-occur. It is likely that many of these climate-related influences will interact at upper slope depths first (200–500 m), where expanding OMZs and deepening acidification effects come in contact. Loss of important deep-water fisheries habitats and thus fishery resources are predicted to result from these climate effects [253, Whitney and Sinclair, unpublished data]. Some clues as to the structure of future ecosystems may be found in OMZs, where low pH and low oxygen occur naturally. In these areas, we see reduced biomass, diversity, and body size, particularly of calcifiers, crustaceans and fishes, whereas squid, jelly fish and annelids do well. Since major changes in temperature, atmospheric CO_2_, oxygen and possibly methane have led to mass extinctions in the past it is likely that significant species loss will occur. However, the speed of current hydrographic change is unprecedented, and thus we enter unknown territory with regard to predicting future changes.

Tangential effects of climate change can include range expansions and contractions associated with changing temperature, as well as oxygen and pH. Increasing temperatures or declining midwater oxygenation may lead species to seek refugia in canyons, on seamounts or down slope. The decrease in depth of the aragonite saturation horizon because of increasing acidification may cause species that rely on this form of calcium carbonate for skeleton formation (e.g. stony corals) to find refuge in the shallower regions of canyons and seamounts [241].

### Invasive species

In shallow waters, the introduction of exotic species leads to major ecosystem-level alterations [285]. The deep sea might at first seem immune to species invasions, but it is not. The red king crab *Paralithodes camtschaticus* was introduced intentionally from the Bering Sea to the Barents Sea to start a fishery. It has expanded along the Norwegian coasts and threatens scallop populations at 300 m [286]. The gastropod *Philine auriformis*, was accidentally introduced from New Zealand to San Francisco Bay in 1993, and made its way to the shelf and upper slope waters (300 m) off southern California, where it forms large populations [287]. Invasive species can be transported by ballast water in tankers, amongst other methods. As an example, the opening of the Suez Canal in 1869 enabled the arrival in the Mediterranean of Indo-Pacific and Erythrean fauna [288]. The limited knowledge of species distributions and identities in the deep sea will make it hard to detect invasions in the future, but there is no question that once they have arrived, successful invaders can change the structure and function of communities [285], [289].

### Synergies and interactions amongst habitats and impacts

**Figure 7:**
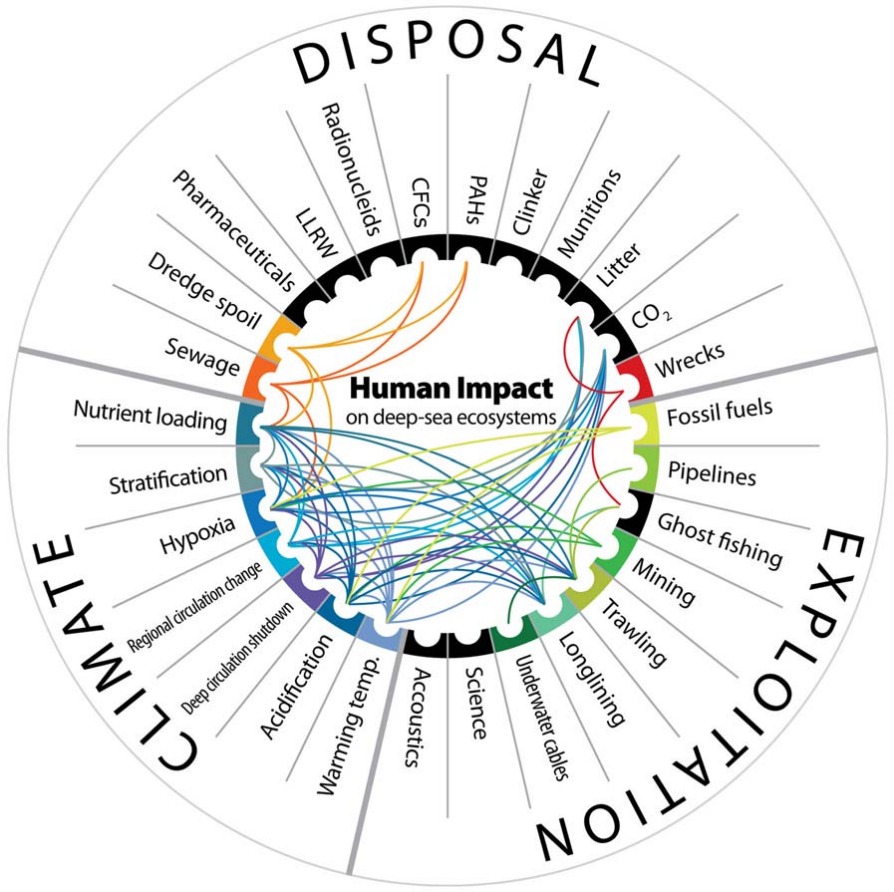
Synergies amongst anthropogenic impacts on deep-sea habitats. The lines link impacts that, when found together, have a synergistic effect on habitats or faunal communities. The lines are colour coded, indicating the direction of the synergy. LLRW, low-level radioactive waste; CFCs, chlorofluorocarbons; PAHs, polycyclic aromatic hydrocarbons.

Although different anthropogenic pressures can have direct effects on deep-sea habitats and fauna, there may also be synergies where two or more impacts interact and have a magnified effect on the ecosystem (Figure 7). Because increased atmospheric CO_2_ and climate change, together with associated effects such as warming, primary production shifts, ocean acidification and hypoxia affect the oceans globally, this is where more synergistic processes will occur, sometimes with positive feedbacks that increase greenhouse effects. Temperature effects on organism tolerances to other stressors are perhaps best understood, although responses are not studied for deep-sea species, or at community or ecosystem levels of organization. For coastal and shelf species, warming temperatures lower oxygen thresholds for many taxa [290] and can reduce tolerance of calcifying species to acidification [170], [291]. Similarly hypoxia and acidification can reduce thermal tolerance windows in marine species, exacerbating the effects of warming [292]. These synergistic interactions would affect all habitats, although those on upper continental margins may be among the first deep-sea environments to experience the confluence of warming, acidification and hypoxia with resource extraction [250], [293]. Commercially fished populations affected in addition by climate-related variations in their habitat might be pushed to levels where the populations cannot be maintained. Global and regional deep-water circulation and ocean stratification will have an effect on the transport of litter, which may accumulate in specific areas. Physical disturbance, imposed by mining, trawling, waste disposal, or oil and gas extraction, tests the resilience of communities weakened by physiological stress from interacting climate factors (temperature, hypoxia or acidification). Altered states, lagged recovery and hysteresis are especially likely outcomes in the deep sea, where rates of recruitment and growth can be slow.

Climate change can cause a shift in the periodicity and intensity of episodic events such as dense shelf water cascading. This can affect local fisheries, as well as intensifying the transport of litter to the deep margin and basins. The accumulation of litter such as plastics or metals can affect the fauna directly by suffocation and causing starvation, but their degradation may also result in the accumulation of microplastics or toxic elements from paints and metals that disrupt hormonal processes of animals.

The disposal of sewage and dredge spoil will add to the effects of hypoxia and nutrient loading related to climate-change, leading to changes in faunal community structure. Wrecks can accumulate litter around them, increasing the possibility of suffocation from plastics, contamination from metal, paints or microplastics and ghost fishing if nets or longlines are tangled around the wreck structure.

Deep-water trawling on habitat builders such as corals will damage structural communities with potential impacts on recruitment and development for other species that use the reefs as nursery and feeding grounds. The effects of trawling on cold-water coral reefs may be even more devastating if acidification of the oceans increases because of climate change. Ocean acidification will probably slow skeletal growth and result in weaker skeletons [238]. Cold-water coral reefs support a significantly higher diversity of species than the surrounding deep-sea floor, but our knowledge on the functional relationships between the frame-building organisms and associated species such as fish and other invertebrates is limited [237] and we do not know the specific effects of ocean acidification on these communities [235]. Furthermore, cold-water corals lack symbiotic zooxanthellae, depending therefore on the input of organic matter from the water column. Changes in surface temperature will change primary productivity and in turn the arrival of organic matter reaching the deep-sea floor that is available for the corals.

### Conclusions: habitats at highest risk (present and future)

The deep sea is clearly not immune from anthropogenic impact. Changes in ocean use, climate and the biodiversity and ecosystem function patterns of deep-sea ecosystems mean that certain habitats are more at risk than others. As resources on land become exhausted, exploitation of the marine environment increases and, with it, so does extraction of the biological and mineral wealth of the deep sea. Furthermore, as the world population grows, the amount of litter produced increases and a large amount finds its way to the oceans and subsequently to the deep seafloor. Long-term anthropogenic pressure will often affect ecosystems at a regional or local scale, but the impact on the wider deep-sea fauna is mostly unknown. Climate change will affect the oceans at a global scale, in some cases amplifying the disturbance caused by other human related activities such as fishing or mining.

**Figure 8:**
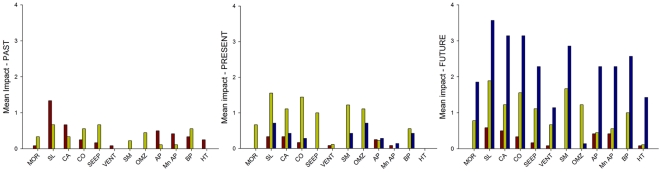
Evolution of the dominant impacts on deep-sea habitats. Mean levels of estimated impact for disposal (red bars), exploitation (green bars) and climate change (blue bars) in past (A), present (B) and future (C) scenarios. Levels of impact estimated from Table 1. MOR, mid-ocean ridge; SL, sediment slope; CA, canyons; CO, corals; SEEP, cold seeps; VENT, hydrothermal vents; SM, seamounts; OMZ, oxygen minimum zones; AP, abyssal plains; Mn AP, manganese nodule abyssal plains; BP, bathypelagic; HT, hadal trenches.

Based on the current knowledge available in the scientific community and expert estimates, we suggest that the overall anthropogenic impact in the deep sea is increasing (Figure 8 A–C, Tables S1, S2 and S3) and has evolved from mainly disposal and dumping in the late 20^th^ century, to exploitation in the early 21^st^ century (Figure 8A & B). At present, exploitation is the most important human-related activity that affects the deep-sea ecosystem, where increasing ecosystem modifications in the future may be caused by climate change (Figure 8B). The habitat types most affected at present, when considering all impacts together, are sediment slopes, followed by cold-water corals, canyons and OMZs (Table S2). Sediment slopes and canyons are mainly affected by fishing, including trawling, longlining and ghost fishing caused by lost or discarded gear. Cold-water corals are especially vulnerable to fishing activities, as the physical damage caused by fishing gear results in the destruction of whole communities of long-lived structural framework builders and associated species. For OMZs, climate change is the most important factor affecting this habitat at present, because of the significant increase in hypoxia. During the remainder of the current century, we predict that the major impact in the deep sea will be climate change (Figure 8C, Table S3), affecting the oceans globally through direct effects on the habitat and fauna as well as through synergies with other human activities. Below we identify the deep-sea habitats that we believe are at higher risk from anthropogenic impact in the future (Table S3):

1. Sedimentary upper slope benthic communities: climate change will have a major impact, particularly caused by the confluence of changes in nutrient input, ocean acidification and spreading of hypoxia. Furthermore, because of the immense global fishing effort on slopes to 1000 m depth, this habitat is, and will be, greatly affected. Although historically these areas have received the most protection from fisheries (e.g. conservation areas), continued efforts to protect vulnerable margin communities against negative impacts of fishing are necessary.
2. Cold-water corals: fishing activities and ocean acidification caused by climate change will be the major impacts affecting cold-water coral communities.
3. Canyon benthic communities: these are mainly affected by fishing activities as improved technologies enable the exploitation of rough terrain such as that found in canyons. Another major impact in canyons will be the accumulation of litter and chemical pollution, accentuated by the conduit effect of canyons and large-scale episodic events such as dense shelf water cascading. Climate change will add pressure to canyon benthic communities by affecting circulation, stratification and nutrient loading.
4. Seamount pelagic and benthic communities: fishing effects on demersal and pelagic species and fishing damage to benthic communities and habitat will greatly affect seamounts, together with changes in global and regional circulation and stratification caused by climate change.

Other ecosystems where future human activities could have a major impact are those with important reserves of mineral resources, such as hydrothermal vents for polymetallic sulphides, manganese nodule abyssal plains, cobalt-rich ferromanganese crusts on seamounts and potential hydrocarbon resources on methane seeps. Although these resources are currently (June 2011) not being exploited, projects for mining massive sulphides from vents are underway and, with the depletion of land-based resources, development of new technologies and the rising price of metals, mining of manganese nodules and cobalt-rich crusts could become commercially viable. Although more distant, pilot programmes for methane hydrate extraction suggest that eventually gas hydrates at seeps will be targeted as an energy source.

There are efforts that aim to lessen the human impacts on the deep sea, such as the establishment of MPAs, marine reserves and no-take zones. Most marine conservation has concentrated on waters lying within the 200-mile exclusive economic zones (EEZs), where successful examples of MPAs and closed areas exist and protect the deep-sea floor. Yet, the EEZs constitute less than 36% of the global ocean. The implementation of regulatory measures in the high seas – 64% of the global ocean – requires a review and changes to the existing UNCLOS legislation to provide wider protection. Because of increased awareness of the vulnerability of deep-sea ecosystems, attitudes have changed considerably and regulatory measures are being introduced wherever legal instruments and authoritative management organizations have been established. Therefore, MPAs and closed areas that protect the deep seafloor and associated vulnerable communities exist both for EEZs and international waters. In the international waters of the Atlantic, the relevant regional fisheries management organizations have recently closed a range of seamount, mid-ocean ridge and slope areas to bottom fisheries. For example, in the Northeast Atlantic Fisheries Commission Regulatory Area of the northeastern Atlantic, such MPAs comprise about 50% of the potential bottom fishing area (i.e. shallower than 2000 m). Other examples include chemosynthetic ecosystems in areas of national jurisdiction in Canada, Portugal, the United States and Mexico that have been partially protected by measures that have been put in place to protect seafloor in general. These are all hydrothermal vents and include the Endeavour Hydrothermal Vents MPA, the Guaymas Basin, the Eastern Pacific Rise Hydrothermal Vents Sanctuary, the US Mariana Trench National Monument in the Pacific Ocean and the Azores Hydrothermal Vent MPA in the Atlantic Ocean. Each of these protected areas follows particular management goals [202]. The Kermadec Benthic Protected area in New Zealand, which includes vent sites, is closed to bottom fishing but the vents are not protected from mining. Furthermore, measures are in place to protect seep communities in the Gulf of Mexico from impact caused by oil and gas extraction, in particular from effects of drilling discharges and anchor placements. However, no protection is in place for large accidents such as the recent (spring 2010) Deepwater Horizon oil spill in the Gulf of Mexico where the effects to the deep-sea fauna were unknown at the time of writing. Areas closed to fishing activities are found in international waters of the Atlantic, Pacific and Indian oceans. Within EEZs there are many examples of protection areas. In New Zealand, almost one third of the New Zealand EEZ is protected from bottom trawling and in the Mediterranean, there is no trawling below 1000 m depth for the entire Mediterranean Sea. Other protected areas exist in Alaska, Hawaii, the Azores and the North Atlantic margin and islands [169].

One of the main problems that continue to cause concern is that the fastest movers in the deep sea are those who wish to use it as a service provider. Lagging behind somewhat are the scientists, managers and legislators. Impacts can occur quickly because they often arise through economic imperatives, while understanding by scientists follows a process governed by funding cycles and with slow and long scientific procedures, thereby introducing a time lag to any response to a perceived threat. Finally, legislators and managers typically act upon concerns raised by evidence (i.e. scientific understanding) and therefore usually follow after science, with the added issue of slow response governed by bureaucratic and political practices that can take years. Human encroachment into the deep sea creates a new conservation imperative. Effective stewardship of deep-sea resources will simultaneously require continued exploration, basic scientific research, monitoring and conservation measures. Each of these activities will benefit from application of basic ecological and conservation theory [293]. As technology offers increasing access to the deep sea, we are provided with opportunities to conduct experiments, generate time series and explore new settings. Where possible, human impacts and protected habitats should be studied as experiments within a regulatory context. Conservation in the deep sea offers challenges in the form of knowledge gaps, climate change uncertainties, shifting jurisdictions and significant enforcement difficulties. With time, technological advances can help address these challenges. It remains to be seen whether new approaches must be developed to conserve the biodiversity and ecosystem services we value in the deepest half of the planet.

## Supporting Information

Table S1 **Expert assessment of past human impacts on the deep sea.** Impacts have been classified from very negative (5) to neutral (0) for each habitat considered. In some cases we have designated no evidence available and an unlikely impact (NA), while in other cases, no evidence is available and potential impact is unknown (?). The total and mean impacts are calculated for disposal, exploitation and climate change in each habitat. A grand total and grand mean are calculated for all impacts affecting each habitat and coded with bold and italics to highlight habitats at major risk. For grand total impact: 0–7 (no format), 8–15 (bold), >16–30 (bold and italics). For grand mean impact: 0–0.4 (no format), 0.5–0.6 (bold), >0.6 (bold and italics). This table was compiled during the SYNDEEP workshop (Scripps, Sept. 2008) with the participation of: Billett DSM, Brand A, Cordes EE, Escobar E, Fournier L, Grassle F, Keller S, Levin LA, Martinez-Arbizu P, Menot L, Metaxas A, Miloslavich P, Priede I, Ramirez-Llodra E, Rowden AA, Sibuet M, Smith CR, Tittensor D, Tyler PA, Vanreusel A, Vecchione M, Snelgrove P, Stocks K. AP, abyssal plains; BP, bathypelagic; Chemical cont. CFCs, chemical contamination by chlorofluorocarbons; Chemical cont. PAHs, chemical contamination by polycyclic aromatic hydrocarbons; CS, cold seeps; CWC, cold-water corals; HT, hadal trenches; HV, hydrothermal vents; MnA, manganese nodules on abyssal plains; MOR, mid-ocean ridges; OMZ, oxygen minimum zones; SC, submarine canyons, SL, sediment slopes; SM, seamounts.
(XLS)

Table S2 **Expert assessment of present human impacts on the deep sea.** For detailed legend see Table S1.
(XLS)

Table S3 **Expert assessment of estimated future human impacts on the deep sea.** For detailed legend see Table S1.
(XLS)
